# Synthesis of Organosilicon Ligands for Europium (III) and Gadolinium (III) as Potential Imaging Agents

**DOI:** 10.3390/molecules25184253

**Published:** 2020-09-16

**Authors:** James I. Bruce, Patrick J. O’Connell, Peter G. Taylor, David P.T. Smith, Roy C. Adkin, Victoria K. Pearson

**Affiliations:** 1 School of Life Health and Chemical Sciences, The Open University, Walton Hall, Milton Keynes MK7 6AA, UK; p.j.oconnell@open.ac.uk (P.J.O.); peter.taylor@open.ac.uk (P.G.T.); d.t.p.smith@open.ac.uk (D.P.T.S.); 2 School of Physical Sciences, The Open University, Walton Hall, Milton Keynes MK7 6AA, UK; roy.adkin@open.ac.uk (R.C.A.); victoria.pearson@open.ac.uk (V.K.P.)

**Keywords:** lanthanide, luminescence, europium, gadolinium, relaxivity, organosilicon, silsesquioxane

## Abstract

The relaxivity of MRI contrast agents can be increased by increasing the size of the contrast agent and by increasing concentration of the bound gadolinium. Large multi-site ligands able to coordinate several metal centres show increased relaxivity as a result. In this paper, an “aza-type Michael” reaction is used to prepare cyclen derivatives that can be attached to organosilicon frameworks via hydrosilylation reactions. A range of organosilicon frameworks were tested including silsesquioxane cages and dimethylsilylbenzene derivatives. Michael donors with strong electron withdrawing groups could be used to alkylate cyclen on three amine centres in a single step. Hydrosilylation successfully attached these to mono-, di-, and tri-dimethylsilyl-substituted benzene derivatives. The europium and gadolinium complexes were formed and studied using luminescence spectroscopy and relaxometry. This showed the complexes to contain two bound water moles per lanthanide centre and T_1_ relaxation time measurements demonstrated an increase in relaxivity had been achieved, in particular for the trisubstituted scaffold 1,3,5-tris((pentane-sDO3A)dimethylsilyl)benzene-Gd_3_. This showed a marked increase in the relaxivity (13.1 r_1p_/mM^−1^s^−1^).

## 1. Introduction

The selective substation and functionalization of aza macrocyclic rings has long been a rich area of synthetic endeavour. This has been due, in no small part, to the extensive use of the macrocycle cyclen (1,4,7,10-tetraazadodecane) as a scaffold to construct ligands for the coordination and complexation for a diverse array of metals, ranging from transition metals, such as zinc, nickel, rhenium, and technetium [1,2,3,4], to the larger lanthanide ions, such as europium, terbium, and gadolinium [5,6,7]. Such complexes attract interest through their potential applications as biomimetic agents, therapeutic agents, molecular sensors, and imaging agents [8,9,10,11]. The primary objective of such derivatization of the macrocycle is to add pendant arms that help define the binding site and act as a means of controlling the properties of the resulting metal complexes. Derivatization, typically at the nitrogen centres, attaches additional functional groups such as carbonyl, ester, alcohol amine and amide functionalities [12,13,14,15] that increase the number and type of donor atoms available for chelation to the central metal ion. This is particularly important for the lanthanide ions, which have high coordination numbers (9–10) and a preference for hard donor atoms such as nitrogen or oxygen. Alkylation using reactive organohalides has been the method of choice for the derivatisation of cyclen and this method has produced ligands for lanthanides, such as europium and terbium, which have been used as the basis for luminescent probes and sensors [8,16,17,18], and gadolinium that has been employed as contrast agents for MRI [11,19,20]. Typically, these synthetic approaches have produced symmetrical tetrasubstituted ligands, although there are examples of tri substituted cyclen derivatives producing coordinatively unsaturated lanthanide complexes for use as anion sensors [21,22]. Examples of disubstituted ligands are rarer, as the resulting complexes lack the required stability in solution [23].

**Figure 1:**
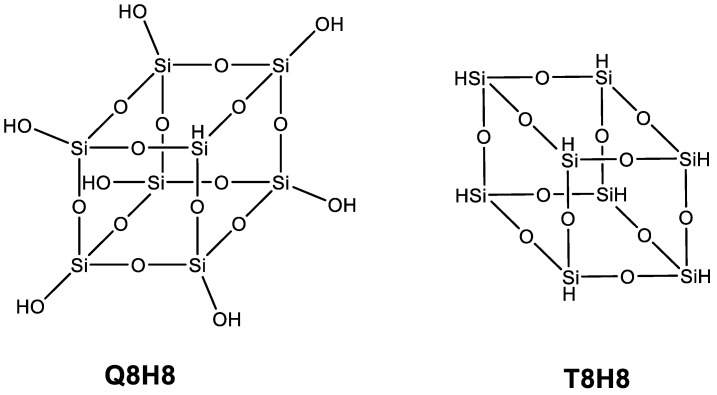
Silsesquioxane cages. The Si-H and Si-OH groups on the silsequioxanes suggested hydrosilylation would be an ideal bond forming reaction between the cages and the complexes and, therefore, the ideal linking functional group on the cyclen ligand would be an alkene. We set out to prepare a range of cyclen ligands that were trisubstituted through secondary carbon centres and contained a fourth arm added subsequently that contained the alkene functionality.

There is a need however to develop alternative strategies for derivatising aza macrocycles, such as cyclen. In particular, the increasing sophistication of targeting probes for diagnostic purposes is driving a need for multifunctional ligands incorporating several functional groups. This is seen in the work of Parker et al. where dicarboxylic acid moieties were attached to cyclen to produce ligands for gadolinium, which included an outer group of carboxylic acid groups. This produced a complex that had an overall negative charge at physiological pH [24]. That synthesis, and those of related ligands, was based on the use of the secondary bromo-derivative of adipic acid. We have also sought to use secondary halides as precursors to form bifunctional ligands for the lanthanide that incorporated additional functionalities [25]. A key attraction is that this would create functional groups on the periphery of the complex that can be used to attach it to larger structures, such as proteins, carbohydrates, and polymers, while limiting any perturbation of the coordination sphere. Building on the synthesis of trisubstituted ligands, we have previously developed a strategy for preparing asymmetrically substituted ligands [26], which can include a non-coordinated linking group within the macrocyclic ring, again, provide a means of adding multiple complexes to a single larger scaffold. This allows luminescent complexes to be readily incorporated into sensors, but equally significantly, it allows gadolinium complexes to be combined to produce multi centre MRI contrast agents with potential high rates of relaxation due to the increase in local concentration and the large size of the macromolecule. This has been demonstrated using polymers such as chitosan [27] but they often had high degrees of internal flexibility, which reduce any potential increases in relaxivity. Dendrimers have proved ideal scaffolds for high molecular weight contrast agents [28]. Silsesquioxanes (Figure 1) are a class of organosilicon oligomer with a well-ordered and defined shape and structure. Moreover, they are readily modified through attachment of pendant arms at the silicon centres [29]. They have previously been used as the core for dendrimer based gadolinium complexes [30] and smaller polydimethylsiloxanes have been used to complex terbium [31]. This suggests they make an ideal anchor for a number of gadolinium complexes.

Our initial attempts focused on the use of secondary halides as the substrate; however, we found, in many cases, the competing elimination reaction became sufficient to render the reaction ineffective. We therefore sought to expand the type of reactions available to us when reacting secondary substrates with azamacrocyclic by exploring the reaction between Michael acceptors and the nitrogen nucleophile in an “aza Michael type” addition reaction. In particular, we examined the reaction involving alkenes substituted with functional groups that made them powerful Michael-type acceptors.

## 2. Results and Discussion

### 2.1. Ligand Synthesis

**Scheme 1:**
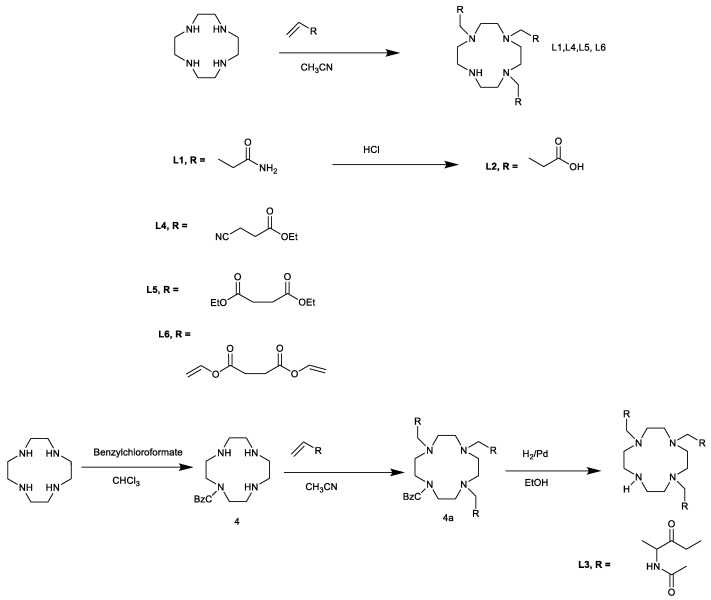
The reaction of cyclen with Michael type donors.

Several methods have been reported for the reaction of a 1,2-disubstituted alkene with a related cyclen analogue, cyclam [32,33,34], giving a range of products from monosubstituted to tetra-substituted macrocycles. We tested the applicability of a Michael type reaction (Table 1) using cyclen and acrylamide involving the refluxing of cyclen in methanol in the presence of a large excess of acrylamide **1**. This resulted in a mixture of tri and tetra substituted products that could be separated using silica chromatography. The major product was the trisubstituted cyclen **L1,** which was obtained in a 9:1 ratio to the tetrasubstituted cyclen. Hydrolysis of the amide function in 12 M HCl acid gave the corresponding carboxylic acid analogue **L2**. We then expanded this to investigate bi functional Michael donors as a means of introducing two functional groups on to the pendant arms of a cyclen ligand (Scheme 1). A reaction between ethyl 2-nitroacrylate **2** (formed from 3-hydroxy -2-nitropropanoate in situ using the method of Babievski [35]) was unsuccessful most probably due to the high reactivity of ethyl 2-nitroacrylate favouring polymerisation under the reaction conditions which involved high temperatures as required for the dehydration. On the other hand, methyl-2-acetamidoacrylate **3** reacted with cyclen; however, only the disubstituted derivative was obtained despite a five-fold excess of the acrylate and refluxing for 24 h. Addition of a catalytic amount of triflic acid allowed the reaction to proceed under room temperature and produced a mixture of tri and tetra substituted products. The use of a CBz protecting group on a single amine position of cyclen allowed us to use **4** to produce the intermediate **4a**. This favoured the trisubstituted derivative and gave the required product **L3** in 70% yield after deprotection. Under similar conditions, ethyl-*cis*-b-cyanoacrylate **5** also produced a trisubstituted derivate **L4** in low yield [36]. It is noteworthy that the addition took place with high regioselectivity at the carbon alpha to the nitrile group. This probably reflects the stronger electron withdrawing ability of the nitrile compared the ester. Methyl 2-acetamidoacrylate **6,** ethyl 3-anilinocrotonate **7**, and ethyl 3-aminocrotonate **8** did not produce significant yields of the trisubstituted products, and only moderate yields of disubstituted products.

In the final case, the Michael acceptor is the alkene in the diallyl maleate **9** or diethyl fumarate **10.** These are excellent Michael acceptors, owing to the electron-withdrawing effect of both carbonyl groups, as has been demonstrated by the reaction of diethyl maleate with secondary amines, such as diethylamine. The reactions were run under identical conditions using acetonitrile as the solvent and potassium carbonate as the base. The dicarbonyl was added in a ratio of 5.4 equivalents to that of cyclen and **L5** was obtained to good yield. Similarly, diallyl maleate was reacted with cyclen to give a trisubstituted product **L6** in moderate yields.

To allow for the attachment of targeting moieties or the incorporation into a larger scaffold or framework, a linking arm was installed on the fourth nitrogen of the cyclen ring via direct alkylation.

**Scheme 2:**
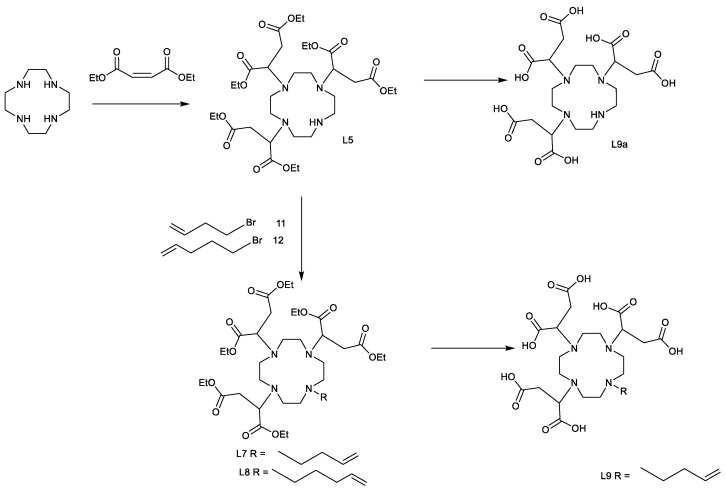
Synthesis of cyclen ligands incorporating dicarboxylic acid pendant arms and an allyl linker.

This involved the reaction of L5 with a suitable bromobutane **11** (Scheme 2) to form butene-L7. The reaction was performed in anhydrous acetonitrile, with potassium carbonate as the base. The use of low temperature reduced the reaction of 4-bromobut-1-ene with itself. A strict 1:1 stoichiometry was also required to prevent a Menshutkin reaction [37], where the tertiary amine was converted to the quaternary ammonium salt through the reaction with a second aliquot of the alkene. We had previously observed these side products in this reaction in the LC–MS and while they could be removed by chromatography, careful control of temperature, and stoichiometry produced a cleaner product.

The success of the reaction could be identified by the presence of alkene resonances in the ^1^H-NMR and the ^13^C-NMR spectra.

An alternative linker arm was based on 5-bromopent-1-ene **12** and was also synthesised to form **L8**. The reaction was performed in anhydrous acetonitrile, to which potassium carbonate and sDO3A-(ethyl) were added. The target compound was identified by using ^1^H-NMR and ^13^C-NMR.

To minimise the risk of decarboxylation when removing the ester groups, lithium hydroxide was used as the base. The resulting lithium salt of **L9** was then converted to the free acid using anion exchange chromatography. The LC–MS confirmed the presence of the L9 ion, and the ^1^H and ^13^C-NMR spectra showed that the ester had been hydrolysed to the acid, by the loss of the protons of the ethyl ester at 1.18 ppm, and the change in ppm of the methylene proton from 4.1ppm to 3.83 ppm in the ^1^H-NMR spectrum. The ^13^C-NMR showed the change in ppm of the carbonyls from 170.93 and 171.46 to 174.9 and 177.3 ppm.

It is worth noting that acid catalysed de-esterification resulted in the loss of one of the pendant arms. Similarly, cation exchange chromatography also resulted in the loss of a pendant arms to a lesser extent.

### 2.2. Organosilicon Scaffolds

The next step was to develop organosilicon scaffolds to allow the incorporation of several complexes into a single molecule. This has the advantage of increasing the local concentration of the sensor, which is useful for analytical applications. There is a further advantage in the gadolinium complexes in MRI applications in that the increased size will slow the rate of tumbling and increase the observed relaxivity. This provides an enhanced contrast in MRI imaging.

**Figure 2:**
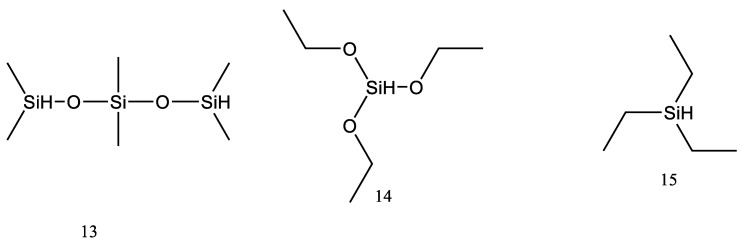
Model organosilicon test compounds.

We tested model reactions using simple siloxanes and silanes (Figure 2).

Three different substrates were chosen: 1,1,3,3,5,5-hexamethyl-trisiloxane **13**, triethoxysilane **14,** and triethylsilane **15**, which contains no Si–O bonds and so should not be susceptible to aqueous decomposition (Figure 2).

The first simple siloxane investigated was triethoxysilane **14**, which was reacted with butene-**L5** in toluene using Speier’s catalyst. The FTIR of the product showed that the Si–H stretch had disappeared, indicating that the hydrosilylation reaction had occurred. The ^1^H and ^13^C-NMR spectra showed a mixture and **L5** and its saturated analogue. The ^29^Si NMR gave only a small signal at −21.38 ppm, showing that majority of the triethoxysilane had decomposed during the reaction.

The experiment was repeated using 1,1,3,3,5,5-hexamethyl-trisiloxane **13** under the same conditions. As with the other reactions, an insoluble residue was formed. The soluble product showed two peaks in the ^29^Si NMR spectra: a major peak at −21.46 ppm, suggesting decomposition had again occurred, and a second peak at −7.79 ppm. The ^29^Si NMR of 1,1,3,3,5,5-hexamethyl-trisiloxane had peaks at −6.20 and −17.30 ppm. The −7.79 ppm peak in the reaction mixture shows that the hydrosilylation reaction had occurred to some extent and that the decomposition product could be a linear polymer. The 1,1,3,3,5,5-hexamethyl-trisiloxane would first be hydrolysed to the silanol, which then reacts with another 1,1,3,3,5,5-hexamethyl-trisiloxane to give 1,1,3,3,5,5,7,7,9,9,11,11-dodecamethylhexasiloxane, which in turn could then react further forming longer chain lengths).

**Figure 3:**
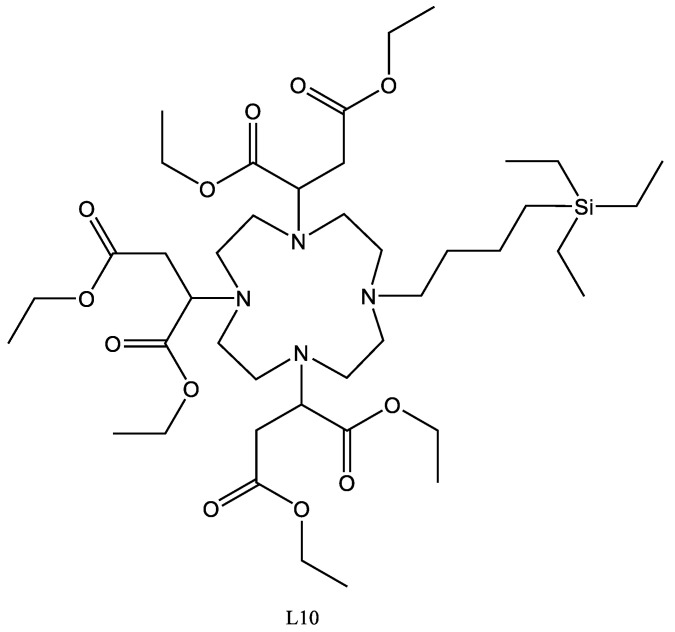
A cyclen based ligand attached to a silicone moiety.

The final test compound was triethylsilane **15**, which is unlike the other test compounds; it is a silane and does not possess a Si–O bond that can be hydrolysed. The reaction was run under the same conditions as before and yielded a soluble clear gum. The loss of the Si–H stretch at 2101 cm^−1^ in the FTIR confirmed complete hydrosilylation. The ^1^H and ^13^C-NMR showed the formation of a new Si–C bond giving peaks at 1.09 ppm and 5.18 ppm respectively (Figure 3). With this success of the hydrosilylation reaction between triethylsilane and **L9** to give compound **L10**, further hydrosilylation was carried out using related silanes.

**Figure 4:**
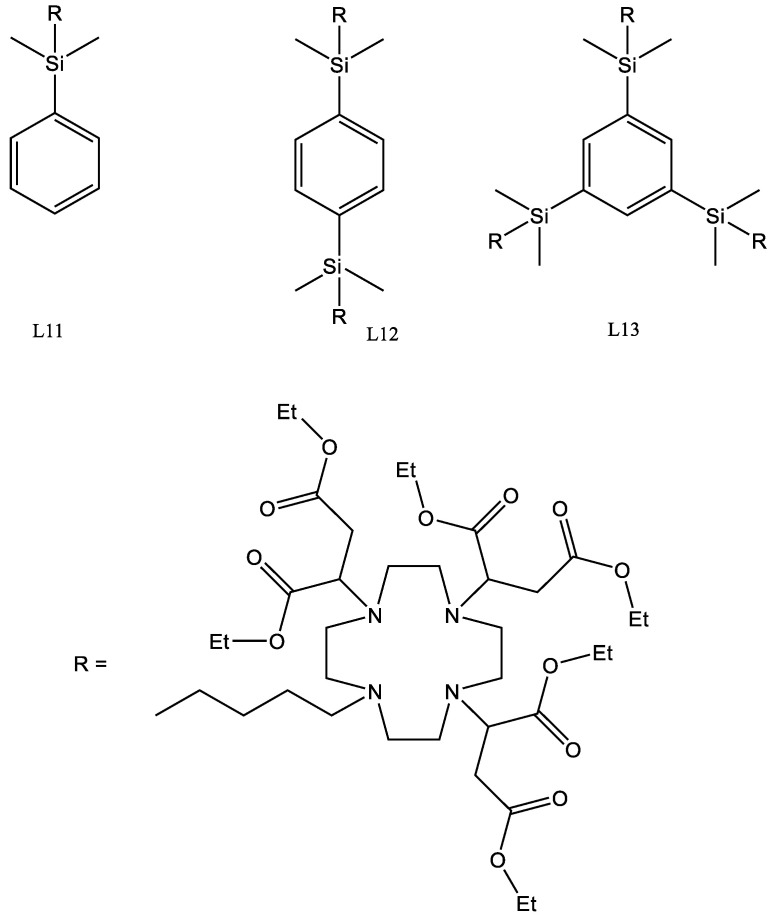
Mono-, di-, and tri- substituted silyl benzenes as scaffolds for cyclen ligands.

To increase the number of complexes in the scaffold, three different substituted benzenes were tested. These were the mono-, di-, and tri-substituted aromatic 1,3,5-silyl benzenes to give ligands **L11**, **L12**, and **L13,** respectively (Figure 4).

**Scheme 3:**
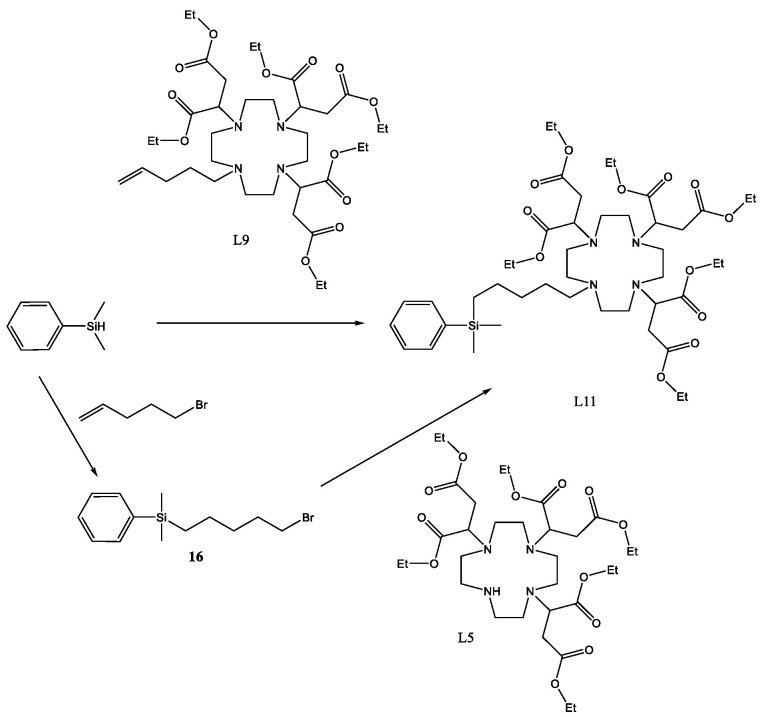
Routes to the synthesis of L11 via cyclen intermediates.

The synthesis was first attempted by the reaction of, 1,3,5, tri-(dimethylsilyl) benzene and **L9** in toluene with Speier’s catalyst. (Scheme 3) The ^1^H, ^13^C, and ^29^Si NMR spectra of the crude product showed that a mixture of products had formed, including some polydimethylsiloxane, as shown by a peak at −21.49 ppm in the ^29^Si NMR.

Multiple signals in the ^29^Si NMR spectra suggested a number of competing reactions had produced a mixture of products that was difficult to purify.

**Scheme 4:**
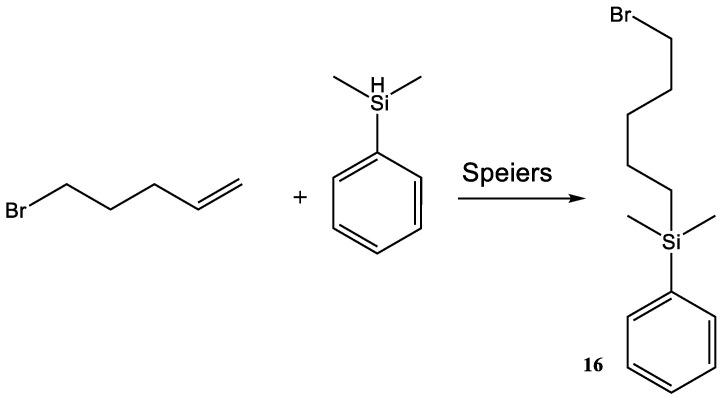
Synthesis of (5-bromopentyl) dimethyl(phenyl)silane.

An alternative route that avoided the decomposition of the **L9**, involved carrying out hydrosilylation reaction between the silane and the alkene before attaching the arm to the **L5**. The first step was the formation of (5-bromopentyl) dimethyl(phenyl)silane **16** by the hydrosilylation reaction between neat dimethyl phenyl silane and an excess of 5-bromopent-1-ene, using Speier’s catalyst (Scheme 4).

**Figure 5:**
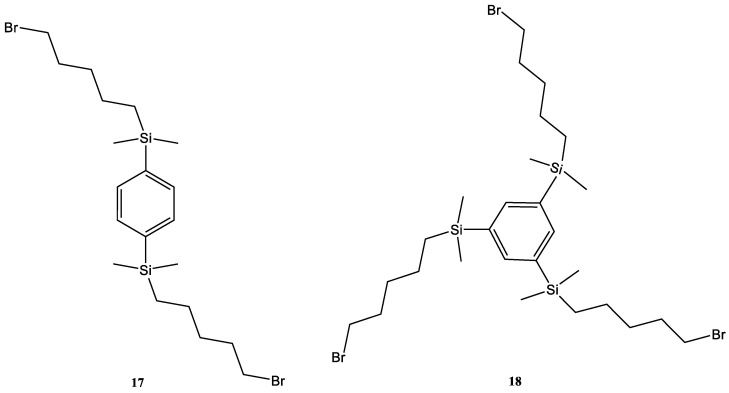
Structures of 1,4-bis((5-bromopentyl) dimethylsilyl) benzene and 1,3,5-tris((5-bromopentyl) dimethylsilyl) benzene.

The product was confirmed by ^1^H and ^13^C-NMR, which showed the loss of the alkene and the formation of the new Si–C bond. The loss of the Si–H was seen in the FTIR and in the ^29^Si NMR, which gave a signal at −2.65 ppm. The formation of both 1,4-bis((5-bromopentyl)dimethylsilyl)benzene **15** and 1,3,5-tris((5-bromopentyl) dimethylsilyl)benzene **16** was achieved using the same reaction conditions, involving 1,4,bis-(dimethylsilyl)benzene and 1,3,5,tri-(dimethylsilyl) benzene, respectively (Figure 5). These products were also confirmed by ^1^H, ^13^C, and ^29^Si NMR and FTIR.

**Scheme 5:**
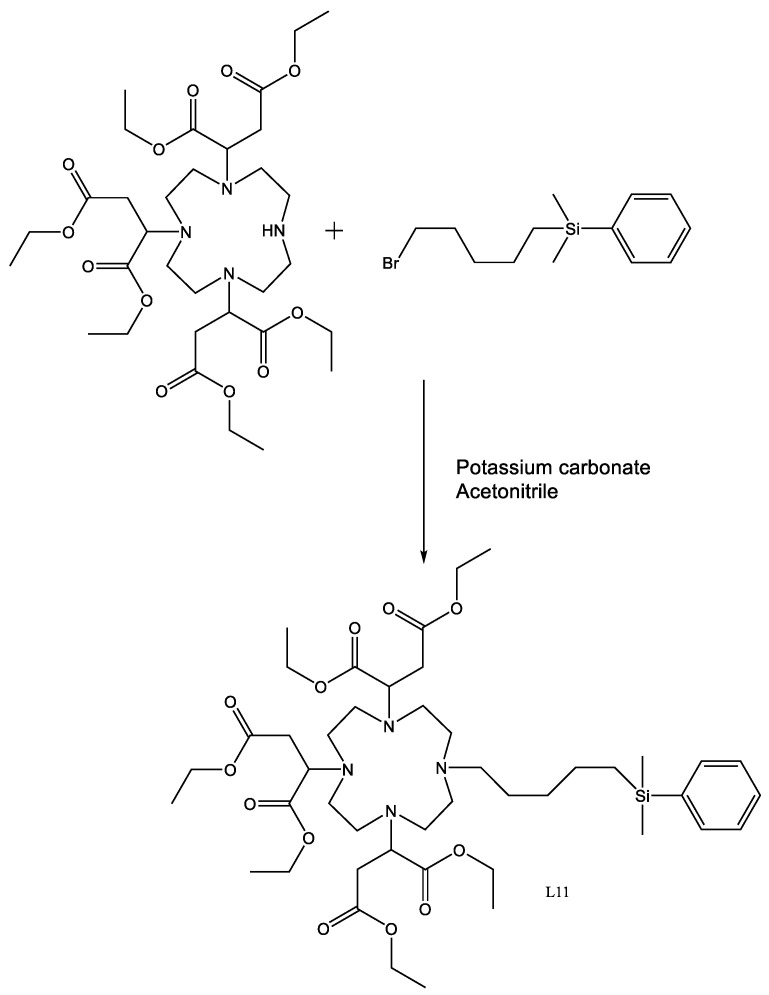
Synthesis of L11 via alkylation.

The second step is the attachment of the **L5** to (5-bromopentyl)dimethyl(phenyl)silane **17**, to form (pentane-sDO3A-(ethyl)) dimethyl(phenyl)silane **L11** (Scheme 5).

**Figure 6:**
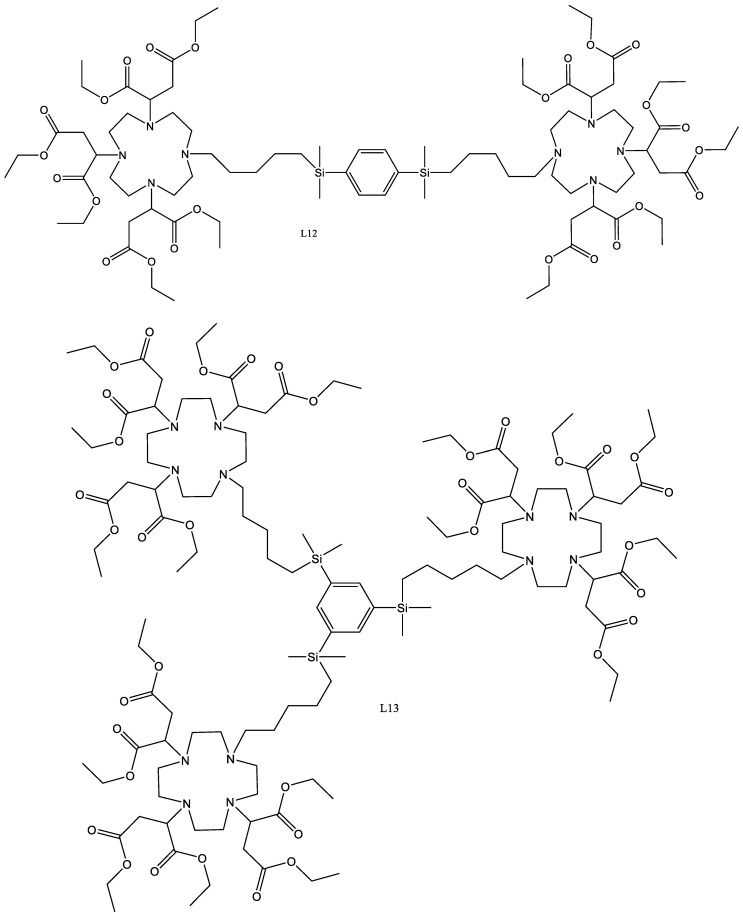
Structures of the bis- (L12) and tris- (L13) substituted scaffolds with cyclen ligands.

**L11** was characterised by ^1^H, ^13^C and ^29^Si NMR spectra and by FTIR and HRMS. The bis and tris reactions followed a similar protocol. The reactions gave yields of around 40%, and the compounds **L12** and **L13** (Figure 6) were characterised by ^1^H, ^13^C and ^29^Si NMR, as well as FTIR and HRMS.

**Scheme 6:**
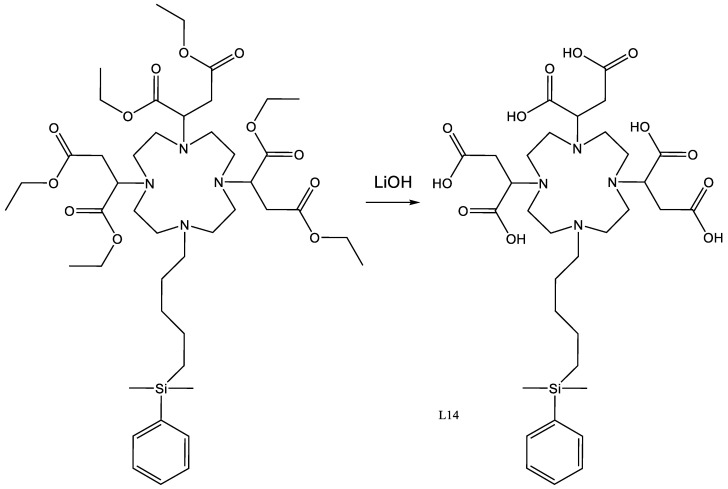
Hydrolysis of the ester groups to produce the target ligands with dicarboxylic pendent arms.

**Figure 7:**
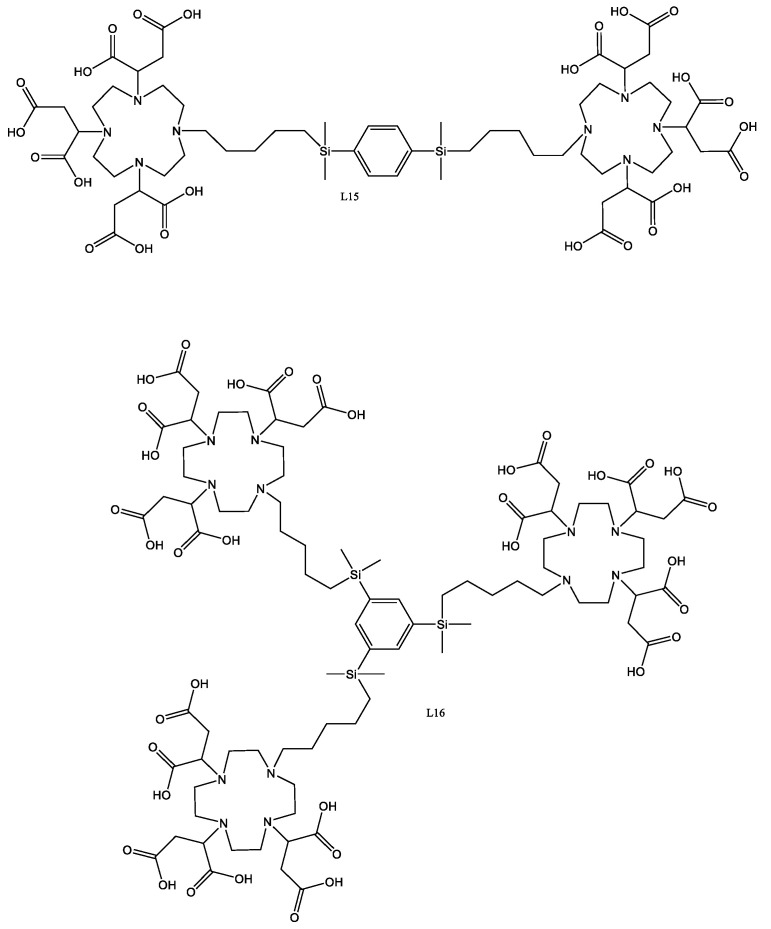
Structures of the bis- (L15) and tris (L16) substituted scaffolds with free acid ligands.

The final step is the removal of the ester protection groups from the ethyl esters. The hydrolysis was achieved by dissolving the ester in 1M LiOH and heating to 100 °C for 24 h (Scheme 6). The ligands **L14** (Scheme 6), **L15**, and **L16** (Figure 7) and were purified by anion exchange chromatography using formic acid (1M).

The presence of the Si groups was identified by the presence of the Si–CH_3_ groups and the protons on the aromatic ring in the ^1^H-NMR.

Mindful of the unsuccessful attempts to react siloxanes with the cyclen ligands via hydrosilylation we tested the methodology on silsesquioxane cages. These are attractive scaffolds due to their large size, rigidity, and the possibility of attaching a high number of lanthanide complexes. The cages T8H8 and Q8H8 were chosen as possible scaffolds.

T8H8 was reacted with the cyclen ligand in the presence of Speier’s catalyst with the steric bulk of the cyclen ligand favouring an α-addition via hydrosilylation. Analysis of the reaction mixture showed the cage had decomposed to PDMS as observed in the Si NMR. Some cyclen ligand was recovered unreacted in combination with the alkane derivative arising from hydrosilylation.

Q8 was reacted using Karstedt’s catalyst and was thought to be less prone to hydrolysis. Again, however significant decomposition via hydrolysis was observed. Further investigation showed that in the presence of the platinum catalyst, a small portion of the cyclen ligand lost a pendant arm and the resulting secondary amine was able to catalyse the hydrolysis of the cages.

The alternative approach of adding the arms first to the cages and then coupling to the cyclen was tried. Moreover, 5-Bromopent-1-ene was added to the Q8M8H cage using Speier’s catalyst and produced the tetrasubstituted product in high yield. Unfortunately, the reaction with the cyclen ligand resulted in decomposition of the cage with the secondary amine again acting as a catalyst for hydrolysis.

### 2.3. Complexation

Ligands **L1**, **L5**, **L7**, **L9a**, **L14**, **L15**, and **L16** were chosen for complexation on the basis of having a range of intermediate single complexes to compare with the larger multicentre complexes based on the organosilicon frameworks. These were dissolved in ethanol or water and solutions of the desired lanthanide—gadolinium or europium—were added as the chloride salt or the oxide. A generic procedure is reported in the experimental while details of the particular complexation reactions can be found in the Supplementary Materials.

### 2.4. Photophysical Studies and Relaxometry

**Figure 8:**
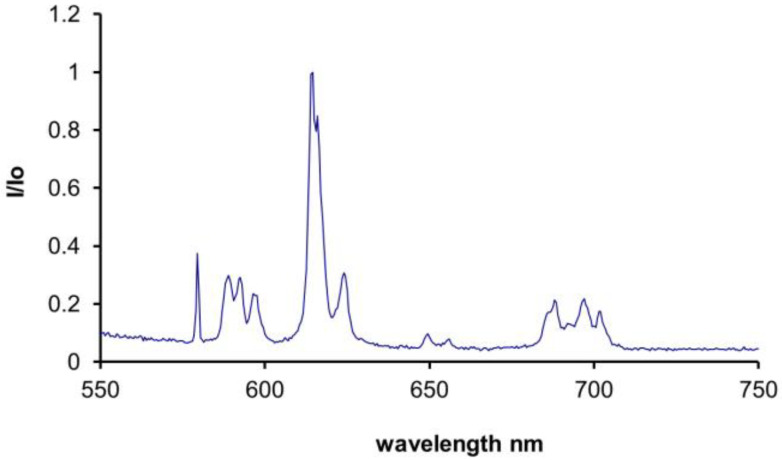
Emission spectrum for EuL14 in H2O λ_ex_ = 265 nm.

The ΔJ = 0 and 1 bands at 570 nm and 580–600 nm, respectively, for the emission spectrum for **EuL14** (Figure 8) are low in intensity, which is indicative of a complex with low symmetry [21,26]. This is also seen in the ΔJ = 2 band at 620 nm, -it is sensitive to the coordination environment and gains intensity due to the limited symmetry of the complex. The ΔJ = 4 at 680–710 nm is also affected by the low symmetry environment, in this case it loses intensity. The three transitions observed for ΔJ = 1 are also typically observed in low symmetry environments. Similar spectra were recorded for **EuL15** and **EuL16**, which suggests each of the europium centres in these di- and trinuclear complexes are in similar coordination environments.

Luminescence lifetimes were recorded in both water and deuterium oxide to allow the determination of the number of bound inner sphere water molecules. Analysis of the rate constants (k) for radiative decay of the Eu ^5^D_0_ excited state in H_2_O and D_2_O allow the calculation of the number of bound waters (q) using established methods [21]. For hexadentate complexes, the hydration state is typically expected to be close to two (Table 2).

The q values are sensitive to the distance of the coordinating water to the europium. This increasing distance reduces the quenching efficiency, which is manifested as a reduction in the q value. The secondary arms of the succinate may hinder close approach of the second bound water molecule and, thus, the observed q values are slightly less that the value of 2 expected for hexadentate complexes. A further decrease observed for ElL14-16 may be attributed to a further increase in hindrance about the Eu centre due to the addition of a bulky forth arm incorporating a phenyl group.

GdCl_3_ and Gd[DOTA] were used as standards to validate the complexation method, analytical technique and calculation methodologies. The results for the Gd[DOTA] were performed at both 25 °C and 20 °C, giving relaxation times of 4.3 r_1p_/mM^−1^s^−1^ and 3.6 r_1p_/mM^−1^s^−1^ respectively, corresponding to known literature values [24]. The relaxivity values of GdL9a and GdL5 were measured, giving 6.4 r_1p_/mM^−1^s^−1^ and 12.6 r_1p_/mM^−1^s^−1^, respectively, while the q values stayed relatively similar at q = 1.68 and q = 1.50. The drop in relaxivity suggests the occurrence of cross-binding, as was reported in earlier studies [26]. Moreover, this may also be borne out by the lifetime measurements, which showed lower q values. The increased concentrations used in the relaxation studies might also increase the extent of cross-binding (Table 3).

The relaxivity of GdL14, with a value of 4.8 r_1p_/mM^−1^s^−1^, is lower than GdL5. This is reflected in the low hydration state of the corresponding Eu complex. It can also be noticed in the comparison between GdL14 and Gd[DOTA] (*q* = 1), which has similar relaxation values, as well as number of coordinated waters. While the relaxivity for GdL14and GdL15 are similar, at 4.8 r_1p_/mM^−1^s^−1^ and 4.5 r_1p_/ mM^−1^s^−1^ respectively, GdL16 shows a marked difference, with a relaxivity of 13.1 r_1p_/mM^−1^s^−1^. This may arise to due intramolecular coordination of adjacent gadolinium metal centres by free carboxylate groups. This in turn would make the complex more internally rigid thus reducing the internal oscillation between the cyclen groups and the aromatic ring. This means that the molecule has only the rotational coefficient to affect the relaxivity, which increases the relaxivity from around 4.5 r_1p_/mM^−1^s^−1^ to over 13 r_1p_/mM^−1^s^−1^. While the increase is a result of the cumulative effect of the gadolinium atoms, it shows the significant effect that the cross-binding has on stabilising the intermolecular movements. The cross-binding effect is not seen in the GdL15, pointing to the gadolinium groups being at a comparatively long distance from each other and therefore being unable to cross-bind. This is reflected in the lower relaxivity of 4.5 r_1p_/mM^−1^s^−1^.

## 3. Materials and Methods

### 3.1. Chemicals

Cyclen (1,4,7,10-tetraazacyclododecane) was purchased from Strem Chemicals, MA, USA. DOTA (1,4,7,10-tetraazacyclododecane-1,4,7,10-tetraacetic acid) was purchased from Sigma-Aldrich. Dry DMF was purchased from Fluka. Dry acetonitrile was purchased from Acros. Potassium carbonate was dried at 105 °C for 1 h before use. 4A and 3A (for alcohols only) molecular sieves were activated in a furnace at 300 °C for 3 h before use. Tetrahydrofuran was distilled from potassium and benzophenone, dioxane was distilled from lithium aluminium hydride, chloroform, and chlorobenzene were distilled from calcium hydride. All solvents were distilled under nitrogen and stored over 4A molecular sieves.

### 3.2. Chromatography

Column chromatography was performed on silica unless otherwise stated. Alumina (Type 507C, neutral) was purchased from Fluka, St Gallen, Switzerland. Thin layer chromatography was carried out on Fluka silica or alumina plates with fluorescent indicator. Thin layer chromatography (TLC) spots were visualised with iodine vapour, UV light, or with potassium permanganate spray (KMnO_4_; water, 0.1% *w*/*v*).

### 3.3. Fluorescence Spectroscopy

Emission spectra and lifetimes were recorded on a FluoroMax P spectrometer JY Horiba, Glasgow, UK.

Corrected emission spectra were recorded using a 420-nm cut-off filter and an excitation wavelength of 397 nm (Eu) excitation, or using sensitized emission at 265 nm. Samples were recorded in aqueous solution with a concentration of 3 mM in 3 mL optically matched cuvettes.

Hydration numbers were calculated using the method of Parker [21] using 0.5 mL optically matched cuvettes with 3 mM solutions of the complexes in H_2_O and D_2_O.

### 3.4. NMR and T1 Relaxivity Measurements

^1^H-NMR and ^13^C-NMR data were obtained using a 300 MHz Bruker spectrometer, Bruker, Covnetry, UK and ^29^Si NMR were recorded on a JEOL 400MHz spectrometer JEOL, Welwyn Garden City, UK. The longitudinal water proton relaxation rates of the complexes were measured at 59.97 MHz and at 25 °C on a Bruker mq 60 NMR analyser. Gadolinium complexes and GdCl_3_ were dissolved in ultra-pure water buffered to pH = 7.5 using MOPS 10 mM.

### 3.5. Mass Spectra

Mass spectra were recorded on a VG Quattro spectrometer, equipped with an electrospray ionization source (ESI). The spectrometer was coupled to a Waters HPLC system. Electrospray was normally used in the positive mode and the samples were diluted in H_2_O: MeOH (50:50). Accurate mass spectrometry was kindly carried out by the Department of Chemistry, University of Wales Swansea, Swansea, SA2 8PP.

Melting points were measured using an electrothermal digital melting point apparatus and are uncorrected. Infrared spectra were recorded as neat films, nujol mulls or KBr discs using a PerkinElmer 1710 infrared Fourier transform spectrometer. Elemental analyses were performed by MEDAC Ltd., Brunel Science Centre, Cooper’s Hill Lane, Englefield Green, Egham, Surrey TW20 0JZ.

## 4. Chemical Characterization

### 4.1. **L1**

Acrylamide (8.9 g, 100 mmol) and cyclen (4.31 g, 25 mmol) were dissolved in 125 mL methanol. The solution was heated under reflux for 24 h. The methanol was removed under reduced pressure and the residue stirred with Et_2_O to give a white paste. The Et_2_O was then decanted and methanol added. After stirring for a few minutes, the product was filtered on a Buchner funnel, washed with ethanol (4 × 10 mL), then Et_2_O. The product was dried in vacuo at 50 °C overnight to give 3.9 as a white solid. Yield: 9.0 g (93%). m.p. 170–172 °C. R*_f_* = 0.57. ^1^H-NMR (300 MHz, D_2_O, δ): 2.29 (6H, t, ^3^J = 6.7 Hz, NCH_2_CH_2_CONH_2_), 2.42 (4H, br s, ring CH_2_), 2.55 (12H, br s, ring CH_2_), 2.62 (6H, t, ^3^J = 6.8 Hz, NCH_2_CH_2_CONH_2_); ^13^C-NMR (75 MHz, D2O, δ); 32.36 (CH_2_CONH_2_), 45.15 (CH_2_), 50.01 (CH_2_), 50.42 (CH_2_,), 50.59 (CH_2_), 51.16 (CH_2_), 51.33 (CH_2_), 178.62 (C = O), 179.11 (C = O).

Anal. Calcd (found) for C_17_H_35_N_7_O_3_*1.0 H_2_O*0.4 C_2_H_5_OH: C, 50.7 (50.5); H, 9.4 (9.1); N, 23.3 (23.5). IR (νmax/(cm-1), KBr): 3349 (NH), 3154 (NH), 2969 (CH), 2837, 1673 (C = O), 1424.

HRMS (+ES): found [M + H] + 386.2878. C17H36N7O3 requires 386.2874.

### 4.2. **L2**

L1 (0.3 g, 0.73 M) was dissolved in 10 mL HCl (12 M) and refluxed vigorously for 2 h. The reaction was switched off and allowed to cool overnight. The white solid, which developed was filtered off and re-dissolved in H2O. After the H_2_O was removed by lyophilisation, the white solid was recrystallised from aqueous methanol. The product was then dried in a vacuum oven at 50 °C for 12 h to give 3.10 as a white solid. Yield: 0.34 g (91.5%). m.p. 180–183 °C. ^1^H-NMR (300 MHz, D_2_O, δ): 2.63 (6H, t, ^3^*J* = 6.6 Hz, CH_2_CH_2_COOH), 3.01 (22 H, br s, CH_2_ NCH_2_CH_2_); ^13^C-NMR (75 MHz, D_2_O, δ); 28.83 (CH_2_), 49.07 (CH_2_), 176.13 (C = O).

Anal. Calcd (found) for C_17_H_32_N_4_O_6_*1.8 H_2_O*2.5 HCl: C, 40.3 (40.7); H, 7.5 (7.1); N, 11.1 (10.9).

IR (ν_max_/(cm^−1^), KBr): 3076 (OH), 1753 (C = O), 1411, 798, 653.

HRMS (+ES): found [M + H] + 389.2395. C_17_H_33_N_4_O_6_ requires 389.2395.

### 4.3. **4**

A solution of benzyl chloroformate (2.7 g, 15.9 mmol) in CHCl_3_ (20 mL, dry) was added slowly over 1.5 h to a cooled solution of cyclen (2.5 g, 14.5 mmol) in chloroform (50 mL). The reaction was allowed to reach ambient temperature and then stirred for 12 h. Some of the organic solvent was removed under reduced pressure and the reaction stirred vigorously after the addition of 5% NaOH (75 mL). The organic layer was separated and the aqueous layer re-extracted with CHCl_3_ (2 × 25 mL). The organic extracts were combined, dried over MgSO_4_ and evaporated in vacuo. The resultant oil was purified on a silica gel column with 1% MeOH in CHCl_3_ containing 0.1% *i*PrNH_2_ to yield an oil which after trituration with acetonitrile provided 10 as a white, crystalline solid (2.6 g, 53%). A small amount of di-Z-cyclen eluted from the column first. *R*_f_ mono-Z-cyclen = 0.51 (SiO_2_, 5% MeOH in CHCl_3_. m.p. 146–152 °C; ^1^H-NMR (300 MHz, CD_3_OD, δ); 3.13 (16H, m, ring CH_2_), 5.15 (2H, s, OCH_2_), 7.36 (5H, m, Ph); ^13^C-NMR (75 MHz, CD_3_OD, δ); 47.87 (ring CH_2_), 48.44 (ring CH_2_), 49.58 (ring CH_2_), 50.51 (ring CH_2_), 68.56 (OCH_2_Ph), 128.89 (3-Ph), 128.93 (4-Ph), 129.08 (2-Ph), 129.37 (1-Ph), 157.52 (C = O): IR (ν_max_/(cm^−1^), KBr): 3391 (NH), 3183 (NH), 2959 (CH), 2837, 2352, 1673 (C = O), 1455, 1012, 776, 728 (arom), 558, 456.

### 4.4. **4a**

The 10 (0.25 g, 0.82 mmol) was dissolved in a 3:1 mixture of CH_3_Cl and CH_3_CN (20 mL). Methyl 2-acetamidoacrylate (0.37 g, 2.61 mmol) was then added. Triflic acid (50 µL, 0.7 eq.) was promptly added with an automatic pipette. This resulted in a clear solution, which was then stirred under argon for 24 h. After the solvent was removed in vacuo, the residue was partitioned between 1 M ammonia solution (10 mL) and chloroform (20 mL). After the aqueous layer was washed with another 10 mL chloroform, the organic extracts were pooled and evaporated to yield a viscous, pale yellow oil. The resulting oil was dissolved in a small amount of CHCl**_3_** and Et**_2_**O was added to induce crystallisation. A colourless, crystalline solid was obtained. Yield: 0.42 g (70.4%). *R_f_* = 0.62 (SiO_2_, 3% *i*PrNH_2_ in CH_2_Cl_2_); m.p. 209 °C (with decomposition); ^1^H-NMR (300 MHz, CDCl_3_, δ): 1.98 (6H, s, CH_3_), 2.06 (3H, s, CH_3_), 2.72–3.25 (20H, m, ring CH_2_), 3.69 (9H, s, CH_3_), 3.78 (2H, s br, NCH_2_CO), 4.55 (m, 3H, CH), 5.80 (2H, s, OCH_2_Ph), 7.24 (5H, m, Ph), 7.68 (br s, 3H, NH); ^13^C-NMR (75 MHz, CDCl_3_, δ); 22.86 (CH_3_), 23.24 (CH_3_), 47.71 (CH_2_), 48.65 (CH), 52.49 (CH_3_), 68.56 (OCH_2_Ph), 127.98 (Ph), 128.69 (Ph), 130.95 (Ph), 170.19 (C = O), 172.19 (C = O).

Anal. Calcd (found) for C_34_H_53_N_7_O_11_: C, 55.50 (55.10); H, 7.26 (7.61); N, 13.32 (13.06). HRMS (+ES): found [M + H] + 736.3871. C_34_H_53_N_7_O_11_ requires 736.3876.

### 4.5. **L3**

The **10a** (0.40 g, 0.54 mM) was stirred in a suspension of 10% palladium on carbon (50 mg) in ethanol (20 mL). A slow flowrate of H_2_ was bubbled through the suspension in a fume cupboard. After 12 h, the reaction mixture was centrifuged to remove the catalyst. The supernatant was filtered through a syringe filter (0.2 µM, polypropylene) and the solvent removed under reduced pressure to give a white solid: [M + H]^+^ = 603). This solid was then heated in 12 M HCl (20 mL) at 60 °C for 3 h. After the acid was removed under reduced pressure, the residue was dissolved in EtOH (20 mL) and treated with activated carbon (0.25 g). The suspension was filtered through a bed of celite on a Buchner funnel and the filtrate was evaporated under reduced pressure. The resulting residue was recrystallised from aqueous EtOH to give **3.31** as a hygroscopic, white solid. Yield: 0.204g (86%). ^1^H-NMR (300 MHz, D_2_O, δ): 1.84 (4H, s, CH_2_), 2.57–3.10 (18H, m, ring CH_2_), 4.60–4.70 (9H, br s, 3NH_3_^+^); ^13^C-NMR (75 MHz, D_2_O, δ); 47.2, 48.6 (CH_2_), 176.61 (C = O).

MS (ES^+^): 434.

### 4.6. **L5**

The 1,4,7,10-tetraazacyclododecane (3 g, 0.01734 mol) and potassium carbonate (7.8 g, 0.05652 mol) were dissolved in anhydrous acetonitrile (100 mL). To this a solution of diethyl maleate (126) (42 g, 0.2439 mol) in anhydrous acetonitrile (100 mL) was added dropwise over 2 h. The reaction was heated to 80 °C and stirred for 72 h. The reaction was cooled to room temperature and the solids were removed. The solvent was removed using reduced pressure to yield a crude oil. The crude extract was chromatographed on silica (Si–60) (350 g), and the product was eluted with DCM/EtOH (95:5).

Yield = 0.25 g, 35.58%.

^1^H-NMR (300 MHz, CDCl_3_, δ): 1.21 (18H, t, ^3^J = 5.01 Hz), 1.80–2.41 (22H, m CH_2_), 3.54 (1H, q, ^3^J = 5.35 Hz CH), 3.77 (4H, q, ^3^J = 7.03 Hz CH_2_), 4.08 (12H, q, ^3^J = 7.01 CH_2_).

^13^C-NMR (75 MHz, CDCl_3_, δ): 14.18 (CH_3_), 35.58 (CH_2_), 46.46 (CH_2_), 47.44 (CH_2_), 50.18 (CH_2_), 50.40 (CH_2_), 59.58 (C = O), 60.52 (CH_2_), 170.93 (C = O), 171.46 (C = O).

FTIR (thin-film, KBr disk): 2983, 1736, 1464, 1368, 1302, 1256, 1170.

HRMS: Theoretical C_32_H_56_N_4_O_12_ 688.3945 Found 711.3792 [M + Na + H].

CHN Theoretical C 55.80 H 8.19 N 8.13 Found C 55.23 H 8.35 N 8.27.

### 4.7. **L6**

Cyclen (390 mg, 2.27 mmol) was dissolved in dry MeCN (35 mL), to which anhydrous potassium carbonate (K2CO3) (1040 mg, 7.51 mmol, 3.3 eq.) was added, heated to 80 °C under an argon atmosphere, and stirred. Added to this mixture, over the course of 1 h, was a solution of diallyl maleate (2.39 mL, 2230 mg, 11.35 mmol, 5 eq.) in dry MeCN (35 mL). The reaction mixture was heated to reflux at 80 °C for 50 h under an argon atmosphere. The organic phase was separated using silica gel chromatography and eluted with a gradient of 1% to 5% EtOH in DCM eluent.

Yield 580mg 33%.

Rf = 0.80.

^1^H-NMR (300 MHz, CDCl_3_, δ): 2.57 (2H, m, CH_2_), 2.73 (2H, m, CH_2_), 2.89 (2H, m, CH_2_), 3.09 (2H, m, CH_2_), 3.84 (1H, m, CH), 4.03 (2H, m, CH_2_) 4.59 (2H, m, CH_2_), 5.19 (2H, m, CH_2_) 5.22 (2H, m, CH_2_), 5.30 (1H, m, CH) 5.34 (1H, m, CH), 5.86 (1H, m, CH).

^13^C-NMR (75 MHz, CDCl3): 34.84 (CH_2_), 47.36 (CH), 47.89 (CH), 50.09 (CH_2_), 51.80 (CH_2_), 57.12 (CH_2_), 61.65 (CH_2_), 65.79 (CH_2_), 66.01 (CH_2_), 66.16 (CH_2_), 118.80 (CH_2_), 119.45 (CH_2_), 119.74 (CH_2_), 131.53 (CH), 131.77 (CH), 170.72 (C = O), 170.80 (C = O), 170.86 (C = O).

HRMS theoretical C_33_H_58_N_4_O_12_ 714.1965 found 714.3542.

CHN Theoretical C 56.12 H 8.20 N 8.11 Found C 57.07 H 8.35 N 8.14.

### 4.8. **L7**

L5 (0.64 g, 0.00093 mol) was dissolved in anhydrous acetonitrile (20 mL) to which potassium carbonate (1.00 g, 0.0072 mol) had been added. The reaction was cooled in an ice/NaCl/IMS bath. To this 4-bromobutene (78) (2.66 g, 0.0197 mol, 2.00 mL) was added and the reaction was allowed to warm up to room temperature overnight. The reaction was then stirred for 78 h at room temperature. The solids within the reaction mixture were removed using a centrifuge, and the solvent was decanted. The solvent was then removed using reduced pressure, followed by 24 h on high vacuum to yield a clear, light-yellow oil. The crude product was then chromatographed on silica eluted with DCM and EtOH (95:5%).

Yield 0.54 g, 0.00072, 77.92%.

^1^H-NMR (300 MHz, CDCl_3_, δ): 1.18 (18H, q, ^3^J = 5.7 Hz CH_3_), 2.09 (2H, d, ^3^J = 6.98 Hz CH_2_), 2.3–2.8 (24H, m, CH_2_), 3.75 (1H, t, ^3^J = 6.0 Hz CH), 4.11 (12H, m CH_2_), 4.93 (2H, q, ^3^J = 17.4 Hz CH), 5.71 (1H, t–t, ^3^J = 6.75 Hz CH).

^13^C-NMR (75 MHz, CDCl3): 14.12 (CH_3_), 14.38 (CH_3_), 29.64 (CH_2_), 35.52 (CH_2_), 49.87 (CH_2_), 50.60 (CH_2_), 51.18 (CH_2_), 53.55 (CH_2_), 53.92 (CH_2_), 54.66 (CH_2_), 59.57 (CH), 59.84 (CH), 59.92 (CH), 60.44 (CH_2_), 60.84 (CH_2_), 115.31 (CH_2_), 137.09 (CH), 171.39 (C = O), 171.89 (C = O).

HRMS: calculated 743.4437 [M + H], found 743.4445 [M + H].

FTIR (thin-film CDCl_3_ NaCl): 2982, 2253, 1723, 1461, 1371, 1300, 1260.

### 4.9. **L8**

sDO3A-(ethyl) (4.06 g, 0.0059 mol) was dissolved in anhydrous acetonitrile (40 mL) to which potassium carbonate (4.1 g, 0.03 mol) was added. The reaction was cooled to −18 °C, then 5-bromopent-1-ene (134) (2.65 g, 0.0177 mol) was added, followed by stirring for a period of 48 h while being kept at room temperature. The solid was removed using a centrifuge, and the solvent layer was decanted. The solvent was then removed using reduced pressure to yield a dark-yellow oil. The crude product was then chromatographed on silica eluted with DCM and EtOH (95:5%).

Yield, 2.77 g, 62%.

^1^H-NMR (300 MHz, CDCl_3_, δ):1.18 (18H, q, ^3^J = 5.6 Hz CH_3_), 2.10 (2H, d, ^3^J = 6.70 Hz CH_2_), 2.3-28 (26H, m, CH_2_), 3.76 (1H, t, ^3^J = 6.0 Hz CH), 4.11 (12H, m, CH_2_), 4.94 (2H, q, 17.5Hz, CH), 5.72 (1H, t–t, ^3^J = 6.75 Hz, CH).

^13^C-NMR (75 MHz, CDCl3): 14.12 (CH_3_), 14.40 (CH_3_), 29.71 (CH_2_), 30.21 (CH_2_), 35.50 (CH_2_), 49.90 (CH_2_), 50.58 (CH_2_), 51.19 (CH_2_), 53.55 (CH_2_), 53.92 (CH_2_), 54.67 (CH_2_), 59.60 (CH), 59.82 (CH), 59.90 (CH), 60.46 (CH_2_), 60.82 (CH_2_), 115.30 (CH_2_), 137.11 (CH), 171.41 (C = O), 171.91 (C = O).

FTIR (thin-film NaCl): 2981, 2940, 2832, 1728. 1448, 1372, 1297, 1258, 1175, 1117.

HRMS: predicted 757.4593 [M + H], found 757.4590 [M + H].

### 4.10. **L9**

L7 (0.25 g, 0.0003355 mol) was dissolved in formic acid (1.4 M, 15 mL), and heated to 60 °C for 48 h. The temperature was increased to 70 °C for 24 h. The solvent was removed using lyophilisation; the solid residue was taken up into water (20 mL) and lyophilised.

Yield 0.21 g, 91%.

^1^H-NMR (300 MHz, CDCl_3_, δ):1.26 (12H, t, ^3^J = 6.01 Hz CH_3_), 2.05 (2H, q, ^3^J = 8.61 Hz CH_2_), 2.54–2.99 (24H, m, CH_2_), 3.77 (1H, q, ^3^J = 3.87 Hz CH), 4.12 (1H, ^3^J = 4.98 Hz CH), 5.02 (2H, q, ^3^J = 8.43 Hz, CH), 5.66 (1H, q, ^3^J = 10.53 Hz, CH).

^13^C-NMR (75 MHz, CDCl3): 14.09 (CH_3_), 35.33 (CH_2_), 44.82 (CH_2_), 48.11 (CH_2_), 49.89 (CH_2_), 61.10 (CH_2_), 63.16 (CH_2_), 116.87 (CH_2_), 134.85 (CH), 170.79 (C = O), 170.81 (C = 0), 170.92 (C = O).

LCMS(postive): 687.81 [M + H] [C_32_H_54_N_4_O_12_]^−^.

FTIR (CDCl_3_–NaCl): 2983, 2858, 2254, 1729, 1609, 1465, 1372, 1299, 1262, 1180.

### 4.11. **L9a**

L5 (0.25 g, 0.00036 mol) was dissolved in ethanol (1 mL), and was added to a solution of formic acid (2.5 mL, 3.05 g, 0.06587 mol) and water (2 mL). The reaction was heated to 90 °C for 24 h, and then cooled to room temperature. The solvents and formic acid were removed using lyophilisation. The lyophilisation was repeated three times by dissolving the crude material in water (10 mL).

^1^H-NMR (300 MHz, D_2_O, δ): 1.31 (4H, m, CH_2_), 2.16–2.35 (15H, m, CH_2_), 2.52 (6H,m, CH_2_), 2.54 (1H, m, CH), 3.42 (2H, m, CH) 4.15 (1H, m, CH).

^13^C-NMR (CD_3_OD): 13.960 (CH_3_), 43.235 (CH_2_), 58.122 (CH_2_), 134.816 (CH), 169.232 (C = O), 175.253 (C = O), 175.944 (C = O), 176.478 (C = O).

HRMS Theoretical C_37_H_64_N_4_O_12_ 756.4582 Found 779.2096 [M + Na].

CHN Theoretical C 58.71 H 8.52 N 7.40 C 58.55 H 8.43 N 7.34.

### 4.12. **L10**

L5 (250 mg, 0.000336 mol) was dissolved in toluene (1 mL), to which triethylsilane (58 mg, 0.0005 mol, 0.08 mL) was added. To the stirred reaction mixture, Speier’s catalyst (20 µL) was added and the reaction was heated to 80 °C for 24 h. The reaction was cooled and passed through an activated charcoal plug. The solvent was then removed using reduced pressure, yielding a clear gum.

Yield 126mg 40%.

^1^H-NMR (300 MHz, CDCl_3_, δ):0.53 (6H, q, ^3^J = 7.86 Hz, CH_3_), 0.98 (9H, t, ^3^J = 5.54 Hz CH_3_), 1.09 (2H, d, ^3^J = 6.03 Hz CH_2_), 1.21 (9H, t, ^3^J = 7.14 Hz), 1.67 (1H, s, CH), 2.28–2.83 (22H, m CH_2_), 3.73 (2H, m CH), 4.09 (2H, q, ^3^J = 4.57 Hz).

^13^C-NMR (75 MHz, CDCl3): 0.93 (CH_3_), 5.75 (CH_2_), 6.51 (CH_2_), 14.02 (CH_3_), 14.35 (CH_3_), 21.35 (CH_2_), 22.57 (CH_2_), 26.59 (CH_2_), 29.13(CH_2_), 29.78 (CH_2_), 35.22 (CH_2_), 35.40 (CH_2_), 49.98 (CH_2_), 50.65 (CH_2_), 51.05 (CH_2_), 51.19 (CH_2_), 53.68 (CH_2_), 53.98 (CH_2_), 55.40 (CH_2_), 59.59 (CH), 60.43 (CH_3_), 171.39 (C = O), 171.44 (C = O), 171.86 (C = O).

^29^Si NMR (CDCl_3_): 19.57, −21.49

FTIR (thin-film NaCl): 3027, 2920, 2874, 1734, 1604, 1495, 1461, 1379, 1177.

HRMS Theoretical C_42_H_78_N_4_O_12_Si 858.5482 Found 858.5865.

CHN Theoretical C 58.71 H 9.15 N 6.52 Found C 59.22 H 9.54 N 6.76.

### 4.13. **16**

Dimethylphenyl silane (1.0 mL, 876 mg, 0.007338 mol) was added to 5-bromopent-1-ene (134) (1.4 eq, 1.04 mL, 1.31 g, 0.008806 mol) along with Speier’s catalyst (100 µL). The reaction mixture was sealed under argon and heated to 60 °C for 34 h. The cooled reaction was added to DCM (150 mL) and activated charcoal was added; the reaction was then refluxed for 30 min. The solids were removed using filtration and the solvent was removed by reduced pressure. The excess 5-bromopent-1-ene was removed using reduced pressure (50 mbar) at 40 °C; the product was then placed under high vacuum for 34 h. The resulting oil was centrifuged to remove trace amounts of activated charcoal, yielding an oil.

Yield 1.23 g, 59.0%.

^1^H-NMR (300 MHz, CDCl_3_, δ):0.03 (6H, s, CH_3_), 0.52 (2H, t, ^3^J = 6.2 Hz CH_2_), 1.13 (4H, m, CH_2_), 1.54 (2H, t, ^3^J = 6.8 Hz, CH_2_), 3.03 (2H, t, ^3^J = 6.9 Hz, CH_2_), 7.07 (3H, t, ^3^J = 3.8 Hz, CH), 7.26 (2H, q, ^3^J = 3.5 Hz, CH).

^13^C-NMR (75 MHz, CDCl3):−1.15 (CH_3_), 15.49 (CH_2_), 23.01 (CH_2_), 31.80 (CH_2_), 32.27 (CH_2_), 33.52 (CH_2_), 127.60 (CH), 129.27 (CH), 133.35 (CH), 139.02 (C).

^29^Si NMR (400 MHz CDCl_3_): −2.65 (Si-R).

FTIR (thin-film NaCl plate): 3068, 2961, 2935, 1642, 1579, 1475, 1252, 1119.

LC–MS (positive): 206.15 [M − Br + H].

### 4.14. **17**

The 1,4-Bis(dimethylsilyl)benzene (1 mL, 875 mg. 0.0045 mol) and 5-bromo-pent-1-ene (2.8 eq, 0.0126 mol, 1.877 g, 1.50 mL) were combined in a sealed tube, to which Speier’s catalyst (100 µL) was added. The reaction was sealed under argon and heated to 60 °C for 48 h. After this step, the reaction was added to DCM (100 mL) and activated charcoal was added; a further stage of reflux for 30 min followed. The solids were filtered out and the solvent removed using reduced pressure, yielding a clear oil. The oil was then placed under high vacuum for 48 h.

Yield 1.37 g, 62.3%.

^1^H-NMR (300 MHz, CDCl_3_, δ):0.01 (12H, s, CH_3_), 0.10 (4H, d, ^3^J = 5.7 Hz CH_2_), 0.49 (4H, t, ^3^J = 6.8 Hz CH_2_), 1.18 (8H, m, CH_2_), 1.53 (4H, p, ^3^J = 7.3 Hz CH_2_), 3.03 (4H, t, ^3^J = 6.9 Hz CH_2_), 7.22 (4H, s CH).

^13^C-NMR (75 MHz, CDCl3): −3.09 (CH_3_), 15.45 (CH_2_), 23.03 (CH_2_), 30.67 (CH_2_), 31.87 (CH_2_), 33.58 (CH_2_), 132.22 (CH), 139.66 (C).

^29^Si NMR (400 MHz CDCl_3_): −2.66. (Si-R).

FTIR (thin-film NaCl plate): 2955, 2926, 1459, 1438, 1379, 1250, 1135, 1056.

LC–MS (positive) 508.1[M + NH4].

### 4.15. **18**

The 1,3,5-tri(dimethylsilyl)benzene (1.00 g, 0.00401 mol), was added to 5-bromopent-1-ene (4 eq, 2.39 g, 0.016 mol, 1.90 mL) along with Speier’s catalyst (100 µL). The reaction was sealed under argon and heated to 60 °C for 48 h. The cooled reaction was added to DCM (100 mL) alongside activated charcoal, and refluxed for 30 min. The solids were removed using filtration and the solvent removed, yielding a clear oil. The oil was then placed under high vacuum for 48 h.

Yield 1.51 g, 54.1%.

^1^H-NMR (300 MHz, CDCl_3_, δ):0.02 (18H, s CH_3_), 0.49 (6H, t, ^3^J = 6.9 Hz CH_2_), 1.14 (H12, m, CH_2_), 1.54 (6H, t, ^3^J = 6.8 Hz, CH_2_), 3.08 (6H, t, ^3^J = 6.8 Hz CH_2_), 7.35 (3H, s, CH).

^13^C-NMR (75 MHz, CDCl3): −3.08 (CH_3_), 15.46 (CH_2_), 23.05 (CH_2_), 31.79 (CH_2_), 32.39 (CH_2_), 33.66 (CH_2_), 137.02 (CH), 139.11 (C_q_).

^29^Si NMR (400 MHz CDCl_3_): −2.59 (Si-R).

FTIR (thin-film NaCl plate): 2957, 2927, 1711, 1460, 1439, 1411, 1367, 1252, 1222, 1138, 1070.

### 4.16. **L11**

L5 (1.15 g, 0.00152 mol) was dissolved in anhydrous acetonitrile (10 mL) to which potassium carbonate (0.90 g, 0.00666 mol) was added along with (5-bromopentyl) dimethyl(phenyl)silane (450 mg, 0.00158 mol). The reaction was sealed under argon and heated to 60 °C for 48 h. The reaction was cooled and the solids removed using a centrifuge. The solvent was then removed using reduced pressure to yield a yellow oil. The crude product was then chromatographed on silica, eluted with a gradient of 100% DCM to 95% DCM + 5% EtOH.

Yield 550 mg, 40.5%.

^1^H-NMR (300 MHz, CDCl_3_, δ):0.20 (6H, s CH_3_), 0.69 (2H, t, ^3^J = 6.4 Hz CH_2_), 1.16 (2H, d, ^3^J = 3.5 Hz CH_2_), 1.19 (6H, t, ^3^J = 2.7 Hz CH_3_), 1.24 (18H, t, ^3^J = 6.9 Hz CH_3_), 2.20 (2H, s, CH_2_), 2.43–2.65 (22H, m, Ch_2_), 3.74 (4H, m CH_2_), 4.10 (12H, q, ^3^J = 7.0 Hz CH_2_), 7.29 (3H, t, ^3^J = 3.3 Hz CH), 7.64 (2H, dd, 2.5 Hz CH).

^13^C-NMR (75 MHz, CDCl3):−3.17 (CH_3_), 14.09 (CH_3_), 14.30 (CH_3_), 15.65 (CH_2_), 18.28 (CH / CH_3_), 23.77 (CH_2_), 26.43 (CH_2_), 31.48 (CH_2_), 35.31 (CH_2_), 49.81 (CH_2_), 50.51 (CH_2_), 51.06 (CH_2_), 53.94 (CH_2_), 55.26 (CH_2_), 58.09 (CH), 59.89 (CH_2_), 60.38 (CH_2_), 127.56 (CH), 128.61 (CH), 133.39 (CH), 139.43 (C), 171.28 (C = O), 171.76 (C = O).

^29^Si NMR (400 MHz CDCl_3_): −2.73. (Si-R).

FTIR: 2922, 2361, 1724, 1634, 1645, 1284, 1252, 1180, 1110, 1088.

HRMS: Theoretical C_48_H_76_N_4_O_12_Si calculated 892.5201, found 893.5286 [M + H].

CHN Theoretical C 60.51 H 8.56 N 6.27 Found C 61.14 H 8.44 N 6.32.

### 4.17. **L12**

L5 (590 mg, 0.0008576 mol) was dissolved in acetonitrile (10 mL) to which potassium carbonate (1.00 g, 0.007407 mol) was added. To the reaction mixture 1,4-bis((5-bromopentyl)dimethylsilyl)benzene (211 mg, 0.0004288 mol) was added and the reaction was sealed under argon and heated to 60 °C for 48 h. The solids were removed using centrifugation and the solvent was extracted by reduced pressure. The crude product was then taken up into DCM (100 mL) to which activated charcoal was added, and then refluxed for 30 min. The solids were removed by filtration and the solvent by reduced pressure to yield a clear oil. The product was then chromatographed on silica eluted with a gradient of DCM (100%) to DCM (95%)/EtOH (5%), yielding the product as an oil.

Yield 303.7 mg, 41.5%.

^1^H-NMR (300 MHz, CDCl_3_, δ):0.22 (12H, s, CH_3_), 0.72 (4H, s, CH_2_), 1.21 (8H, m CH_2_), 1.26 (36H, m CH_3_), 1.9 (4H, s, CH_2_), 2.23–2.75 (48H, m, CH_2_), 3.71 (6H, m, CH_2_), 4.07 (24H, m CH_2_), 7.49 (H4, s, CH).

^13^C-NMR (75 MHz, CDCl3):−3.20.

(CH_3_), 13.09 (CH_3_), 14.80 (CH_3_), 16.25 (CH_2_), 18.48 (CH / CH_3_), 24.07 (CH_2_), 26.43 (CH_2_), 31.48 (CH_2_), 35.31 (CH_2_), 49.81 (CH_2_), 50.51 (CH_2_), 51.06 (CH_2_), 53.94 (CH_2_), 55.26 (CH_2_), 58.09 (CH), 59.89 (CH_2_), 60.38 (CH_2_), 127.56 (CH), 128.61 (CH), 133.39 (CH), 138.44 (C_q_), 171.22 (C = O), 174.06 (C = O).

^29^Si NMR (400 MHz CDCl_3_): −2.85 (Si-R).

FTIR (thin-film NaCl plates): 2983, 2930, 1724, 1465, 1447, 1371, 1302, 1260, 1170, 1031, 909.

HRMS: Theoretical C_84_H_146_N_8_O_24_Si_2_ 1707.0921 Found 1707.7698.

CHN Theoretical C 59.06 H 8.61 N 6.56 Found C 58.86 H 8.91 N 6.23.

### 4.18. **L13**

The 1,3,5-bis((5-bromopentyl)dimethylsilyl)benzene (674.5 mg, 0.00109 mol), was dissolved in acetonitrile (10 mL) to which L5 (2.25 g, 0.00327 mol) was added, along with potassium carbonate (2.20 g, 0.016 mol). The reaction was heated to 60 °C for 48 h under argon, after which the solids were removed using centrifugation and the solvent was removed using reduced pressure. The crude oil was then taken up into DCM (100 mL) to which activated charcoal was added. The reaction was refluxed for 30 min, and the solids were removed using filtration. The solvent was then removed using reduced pressure to yield the crude product. The oil was chromatographed on silica eluted with a gradient of DCM (100%) to DCM (95%) EtOH (5%) leaving a clear oil.

Yield: 1.18 g, 43.0%.

^1^H-NMR (300 MHz, CDCl_3_, δ):0.33 (18H, s, CH_3_), 0.72 (6H, m, CH_2_), 1.37 (72H, m CH_3_), 2.36–2.71 (72H, m, CH_2_), 3.88 (9H, m, CH_2_), 4.12 (36H, m CH_2_), 7.69 (3H, s, CH).

^13^C-NMR (75 MHz, CDCl_3_):−3.13 (CH_3_), 13.98 (CH_3_), 14.25 (CH_3_), 15.68 (CH_2_), 18.19 (CH_3_), 23.90 (CH_2_), 25.96 (CH_2_), 28.12 (CH_2_), 31.66 (CH_2_), 35.52 (CH_2_), 35.17 (CH_2_), 35.31 (CH_2_), 46.58 (CH_2_), 47.83 (CH_2_), 51.81 (CH_2_), 51.06 (CH_2_), 53.70 (CH_2_), 57.92 (CH_2_), 58.54 (CH), 59.88 (CH), 60.33 (CH_2_), 60.37 (CH_2_), 61.50 (CH_2_), 137.23 (CH), 139.05 (C_q_), 171.43 (C = O), 178.82 (C = O).

FTIR (flush sample CDCl_3_, NaCl plate): 2981, 2932, 2854, 2360, 2258, 1729, 1448, 1372, 1299, 1254, 1175, 1030.

^29^Si NMR (400 MHz CDCl_3_): −2.83 (Si-R).

MALDI: Theoretical C_123_H_216_N_12_O_36_Si_3_ 2521.4 Found 2521.7.

CHN Theoretical C 58.55 H 8.63 N 6.66 Found C 59.86 H 8.71 N 6.73.

### 4.19. **L14**

L11 (0.55 g, 0.0006162 mol) was dissolved in 1M LiOH (5 mL) and heated to 100 °C for 24 h. The solution was cooled to room temperature, then was injected into the head of the ion-exchange column containing Bio-Rad AG1 A4I (OH− form), bed volume 500 cm^3^, eluting with ultra-pure water at a rate of 5 mL/min. After collection of 500 mL of eluent, the eluent was changed to formic acid (1 M), while the fraction was collected in 5 mL units and analysed using negative-ion LC–MS. The solvent was removed by lyophilisation to yield a white solid, which was then dissolved in water (10 mL) and the lyophilisation repeated to produce a white solid.

Yield 143 mg, 32%.

^1^H-NMR (300 MHz, D_2_O δ): 0.01 (6H, s, CH_3_), 0.53 (2H, s, CH_2_), 1.09 (4H, s, CH_2_), 1.43 (2H, s, CH_2_), 2.11–2.79 (24H, m, CH_2_), 3.83 (3H, m, CH), 7.17 (3H, m CH), 7.39 (2H, m, CH).

^13^C-NMR (75 MHz, D_2_O):−3.21 (CH_3_), 23.77 (CH_2_), 28.03 (CH_2_), 33.52(CH_2_), 36.36 (CH_2_), 48.61 (CH_2_), 50.67 (CH_2_), 51.30(CH_2_), 54.04 (CH_2_), 55.16 (CH_2_), 57.89 (CH), 59.89 (CH_2_), 60.38 (CH_2_), 129.03 (CH), 131.52 (CH), 135.11 (CH), 137.21 (C_q_). 174.68 (C = O), 179.86 (C = O).

^29^Si NMR (400 MHz D_2_O): −2.80 (Si-R).

FTIR (KBr disk): 3012, 2880, 2583, 1726, 1512, 1485, 1376, 1327, 1296.

HRMS: Theoretical C_33_H_52_N_4_O_12_Si 724.3487 Found 747.5834 M + Na.

CHN Theoretical C 54.68 H 7.23 N 7.73 Found C 55.86 H 7.71 N 7.13.

### 4.20. **L15**

L12 (1.72 g, 0.001 mol) was dissolved in LiOH (5 mL) and heated to 100 °C for 24 h. The solution was cooled to room temperature, then was injected into the head of the ion-exchange column containing Bio-Rad AG1 A4I (OH− form), bed volume 500 cm^3^, eluting with ultra-pure water at a rate of 5 mL/min. After collection of 500 mL of eluent, the eluent was changed to formic acid (1M), while the fraction was collected in 5 mL units and analysed using negative-ion LC–MS. The solvent was removed from the product-containing fractions by lyophilisation to yield a white solid, which was then dissolved in water (10 mL) and the lyophilisation repeated to give a white solid.

Yield, 629.1 mg, 45.9%.

^1^H-NMR (300 MHz, D_2_O δ): 0.02 (12H, s, CH_3_), 0.89 (4H, s CH_2_), 1.06 (4H, s, CH_2_), 1.09 (4H, s, CH_2_), 2.47–3.89 (50H44, m, CH_2_), 7.62 (4H, m, CH).

^13^C-NMR (75 MHz, D_2_O):− 3.19(CH_3_), 23.77 (CH_2_), 28.03 (CH_2_), 33.52(CH_2_), 36.36 (CH_2_), 48.61 (CH_2_), 50.67 (CH_2_), 51.30(CH_2_), 54.04 (CH_2_), 55.16 (CH_2_), 57.89 (CH), 59.89 (CH_2_), 60.38 (CH_2_), 129.03 (CH), 131.52 (CH), 135.11 (CH), 137.21 (C_q_). 174.68 (C = O), 179.86 (C = O).

^29^Si NMR (400 MHz D_2_O): −2.82 (Si-R).

FTIR: 3218, 3052, 2953, 2576, 1927, 1725, 1631, 1580, 1467, 1392, 1248, 1187, 1135, 1086.

HRMS: Theoretical C_60_H_98_N_8_O_24_Si_2_ 1370.6282 Found 1371.7254 M + H.

CHN Theoretical C 52.54 H 7.20 N 8.17 Found C 52.34 H 7.41 N 8.13.

### 4.21. **L16**

L13 (2.00 g, 0.000788 mol) was dissolved in LiOH (25 mL) and heated to 100 °C for 48 h, forming a colloidal solution. The solution was filtered through a 5 µm glass-fibre filter pad, then the solution was neutralised using formic acid (1M). The solution was lyophilised to form a white solid, which was then taken up into LiOH (1M) (50 mL) forming a clear solution. The solution was injected into the head of the ion-exchange column containing Bio-Rad AG1 A4I (OH− form), bed volume 500 cm^3^, eluting with ultra-pure water at a rate of 5 mL/min. After collection of 500 mL of eluent, the eluent was changed to formic acid (1M), while the fraction was collected in 5 mL units and analysed using negative-ion LC–MS The solvent was removed by lyophilisation to yield a white solid, which was then dissolved in water (10 mL) and lyophilisation was repeated.

Yield 283.2 mg, 17.65%.

^1^H-NMR (300 MHz, D_2_O δ): −0.05 (H18, s), 0.48 (H6, s), 0.87 (H6, s), 1.15 (H6, s), 1.60 (H6, s), 2.35–3.03 (H72, m), 3.93 (H9, m), 7.46 (H3, s).

^13^C-NMR (75 MHz, D_2_O):− 3.28 (CH_3_), 23.77 (CH_2_), 28.03 (CH_2_), 33.52(CH_2_), 36.36 (CH_2_), 48.61 (CH_2_), 50.67 (CH_2_), 51.30(CH_2_), 54.04 (CH_2_), 55.16 (CH_2_), 57.89 (CH), 59.89 (CH_2_), 60.38 (CH_2_), 129.03 (CH), 131.52 (CH), 135.11 (CH), 137.21 (C_q_). 174.68 (C = O), 179.86 (C = O).

^29^Si NMR (400 MHz D_2_O): −2.83 (Si-R).

FTIR (KBr disk): 3402, 3132, 2975, 2858, 1734, 1639, 1597, 1384, 1251, 1179.

MALDI: Theoretical C_87_H_144_N_12_O_36_Si_3_ 2016.9 Found 2018.2.

CHN Theoretical C 51.77 H 7.19 N 8.33 Found C 52.84 H 7.51 N 8.15.

### 4.22. **GdL14**

Dimethyl(phenyl)silane-pentyl-sDO3A (11.3 mg, 0.0000157 mol) was dissolved in water (10 mL) to which Gd2O3 (2.8 mg, 0.00000785 mol) was added. The pH of the solution was adjusted from pH 5 to pH 8–8.5 by addition of LiOH (1M) solution. The reaction was then heated to 80 °C for 48 h. The solution was cooled and the solids removed using a centrifuge, then the solution was decanted and passed through a 0.5 µm filter. The solution was lyophilised, producing a white powder.

Yield 10.1 mg, 73.1%.

FTIR (KBr disk): 3415, 2954, 2932, 2867, 2359, 1587, 1392, 1317, 1252, 1182, 1153, 1115, 1084.

CHN Theoretical C 41.49, H 5.81, N 5.61 found C 41.15 H 5.34, N 6.05.

### 4.23. **EuL14**

(pentane-sDO3A-) dimethyl(phenyl)silane (26.7 mg, 0.000031 mol) was dissolved in water (20 mL) to which Eu2O3 (5.5 mg, 0.0000155 mol) was added. The pH was adjusted from pH 5 to pH 8.5 using LiOH and the reaction was heated to 80 °C for 48 h. The solution was centrifuged and then passed through a 0.5 µm filter, after which it was lyophilised, producing a white powder.

Yield 22.5 mg, 83%.

FTIR (KBr disk): 3417, 2954, 2932, 2867, 2359, 1588, 1393, 1317, 1253, 1184, 1152, 1115, 1083.

CHN Theoretical C 40.89, H 5.79, N 5.66 found C 41.38 H 5.84, N 6.12.

## 5. Conclusions

The use of an aza Michael type reaction is a reasonable alternative to alkylation particularly when adding branched pendant arms, i.e., when cyclen is reacting with a secondary carbon centre. It is however not a complete success and the ease and efficacy of the reaction is influenced by the nature on the groups on the Michael donor, with strongly electron withdrawing groups generally promoting a complete reaction. While the complex structures of the silsesquioxane cages could not survive the hydrosilylation conditions in the presence of the cyclen ligands, the simpler dimethylsilyl benzenes were robust and produced large ligand scaffolds able to coordinate the lanthanide centres and form stable complexes.

## Figures and Tables

**Table 1:** Michael Donors used in reaction with cyclen. Yields are in parentheses.

Donor (% Yield)	
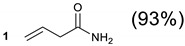	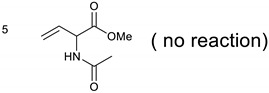
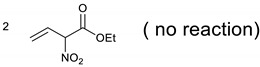	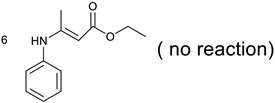
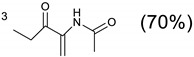	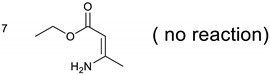
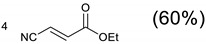	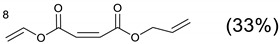
	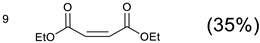

**Table 2:** Radiative decay constants and hydration numbers for Europium complexes.

Complex	k_H2O_	k_D2O_	q
EuL7	3.457	1.81	1.49
EuL5	3.423	0.78	1.5
EuL9a	4.563	1.75	1.68
EuL14	1.758	0.78	0.75
EuL15	1.95	0.769	0.97
EuL16	1.894	0.772	0.9

**Table 3:** Relaxivity values for Gadolinium complexes.

Complex	r_1p_/mM^−1^s^−1^
GdCl_3_	8.6
GdDOTA	4.3
GdL1	4.6
GdL7	5.7
GdL5	12.6
GdL9a	6.4
GdL14	4.8
GdL15	4.5
GDL16	13.1

## Supplementary Materials

Additional data for chemical characterization of the following: GdL1, GdL7, GdL5, EuL5, GdL9a, EuL9a, GdL15, EuL15, GdL16, EuL16.
