# Supplementary material for: Synthesis of Organosilicon Ligands for Europium (III) and Gadolinium (III) as Potential Imaging Agents
Source: Molecules. 2020 Sep 16;25(18):4253. doi: 10.3390/molecules25184253 (PMC7570700; doi:10.3390/molecules25184253)
Supplement: Supplementary file 1 [file molecules-25-04253-s001.pdf]

Supplementary information

Dimethyl chlorosilane (37.60 g, 0.3968 mol) was dissolved in THF (120 ml) to which magnesium turnings (9.68 g, 0.3968 mol) were added and stirred until dissolved. The solution was then added dropwise to a slowly refluxing solution of 1,3,5-tribromobenzene (25 g, 0.078 mol) dissolved in THF (80 ml). The reaction was then refluxed for 3 hours, after which the solvent was removed using reduced pressure to yield an orange solid. The product was extracted using hexane (4 x 50 ml); the organic phases were combined and filtered and the solvent removed using reduced pressure, yielding a crude oil. The oil was then distilled under high vacuum and a product collected at 60–65oC.

Yield 6.98 g,, 35.5%

FTIR (thin-film NaCl plates): 3004, 2960, 2902, 2122, 1558, 1426, 1380, 1365, 1249

HRMS calculated 250.1102 , found 251.1104 [M+H]

1H NMR (CDCl3): 0.24 (18H, t, ^3J =2.7 Hz CH_3), 4.34 (3H, q, ^3J =3.7Hz Si-H), 7.63 (3H, s CH)

13C NMR (CDCl3): −3.72 (CH3), 136.75 (CH), 140.54 (C_q).

29Si NMR (CDCl3): −15.99

GdL1

L1 (0.15 g, 0.39 mmol) was suspended in anhydrous methanol under an inert atmosphere. Addition of GdCl3 (anhydrous) to 3.2 caused it to become fully-soluble. The solution was heated at 60 C overnight under argon. After the reaction was stopped, most of the MeOH was removed at 40 C under reduced pressure. Et2O was added dropwise until turbidity just began. After cooling in the freezer overnight, the white, dense precipitate (4.4) which developed was collected by centrifugation. Yield: 0.19 g (70 %). m.p. > 250 °C.

Rf = 0.4 (Silica plate, acetonitrile-water-tBuOH, 2:9:2 v/v).

HRMS (+ES): found [M+2(NH4Cl)+Cl+H]+ 681.1776. [M+2(NH4Cl)+Cl+H]+ requires 681.1775.

GdL7

L7 (600 mg, 0.00081 mol) was dissolved in EtOH (3 ml), to which GdCl3 (180 mg, 0.00068 mol) was added. The reaction was refluxed for 48 hours. The cooled reaction was added to ice-cold diethyl ether (25 ml). The solid formed was collected by centrifuge and washed with cold diethyl ether (2 x 50 ml) and dried under vacuum. The solid was taken up into methanol (1 ml) and room-temperature diethyl ether added until precipitation occurred. The sample was then cooled to −18oC for 18 hours. The solid formed was collected by centrifugation.

Yield 180 mg, 24.6%. Decomposes at 275 oC.

FTIR (KBr disk): 3376, 2360, 1732, 1615, 1440, 1242.

CHN: $C_{36}H_{62}N_4O_{12}Gd$ Theoretical C 48.04 H 6.94 N 6.22 Found C 47.87, H 6.62, N, 6.38

EuL7

L7 (370 mg, 0.00049 mol) was dissolved in EtOH (3 ml), to which EuCl3 (118 mg, 0.000456 mol) was added. The reaction was refluxed for 48 hours. The cooled reaction was added to ice-cold diethyl ether (25 ml). The solid formed was collected by centrifuge and washed with cold diethyl ether (2 x 50 ml) and dried under vacuum. The solid was taken up into methanol (1 ml) and room-temperature diethyl ether added until precipitation occurred. The sample was then cooled to −18 oC for 18 hours. The solid formed was collected by centrifugation.

Yield 109 mg, 0, 24.8%

FTIR (KBr disk): m 2268, 2984, 2360, 1728, 1641, 1467, 1278, 1179.

MADLI-MS: Predicted 757.92 . Found 757.4

GdL5

L5 (400 mg, 0.00058 mol) was dissolved in EtOH (2 ml) to which GdCl3 (139 mg, 0.00051 mol) was added. The reaction was refluxed for 24 hours. The cooled reaction was added to ice-cold diethyl ether (25 ml). The solid formed was collect3ed by centrifuge and washed with cold diethyl ether (2 x 50 ml) and dried under vacuum. The solid was taken up into methanol (1 ml) and room-temperature diethyl ether added until precipitation occurred. The sample was then cooled to −18 oC for 18 hours. The solid formed was collected by centrifugation.

Yield: 227 mg, , 46.3%.

FTIR (KBr disk): 3397, 1729, 1594, 1439, 1371, 1302, 1179, 1089.

CHN: $C_{32}H_{56}N_4O_{12}Gd$ Theoretical C 45.48 H 6.56 N 6.63 Found C 45.56, H 6.41, N, 6.72

EuL5

L5 (457 mg, 0.00069 mol) was dissolved in EtOH (2 ml) to which EuCl3 (162 mg, 0.000627 mol) was added. The reaction was refluxed for 24 hours. The cooled reaction was added to ice-cold diethyl ether (25 ml). The solid formed was collected by centrifuge and washed with cold diethyl ether (2 x 50 ml) and dried under vacuum. The solid was taken up into methanol (1 ml) and room-temperature diethyl ether added until precipitation occurred. The sample was then cooled to −18 oC for 18 hours. The solid formed was collected by centrifugation.

Yield: 356 mg, 75.4%.

FTIR (KBr disk): 3429, 2920, 2857, 2360, 1601, 1441, 1314, 1268, 1185, 1153, 1094.

CHN: $C_{32}H_{56}N_4O_{12}Eu$ Theoretical C 45.77 H 6.60 N 6.67 Found C 45.37, H 6.21, N, 6.98

GdL9a

.L9a(108 mg, 0.0002105 mol) was dissolved in HCl solution (2 ml, pH 3) to which Gd2O3 (37.8 mg 0.000105 mol) was added. The reaction was then heated to 80 oC for 24h, after which it was allowed to cool, and then passed through a 5 μm filter. The resulting filtrate was freeze-dried, producing a white solid.

Yield 112 mg, 78.8%

FTIR (KBr disk): 3229, 2981, 2830, 2254, 1631, 1415, 1368, 1311, 1274, 1222, 1204, 1174.

CHN Theoretical C 23.50, H 3.24, N 5.48 found C 23.17, H 3.69, N 5.1

EuL9a

L9a (20 mg, 0.0000384 mol) was dissolved in water (5 ml) to which to which Eu2O3 (6.8 mg 0.0000194 mol) was added. The reaction was then refluxed for 24 hours, after which it was allowed to cool, and then passed through a 5 μm filter. The resulting filtrate was freeze-dried, producing a white solid.

Yield 21 mg, , 81.5%

FTIR (KBr disk): 3420, 2975, 2921, 2884, 1720, 1592, 1413, 1186, 1091

MALDIMS: Predicted 743.91 Found 743.40

GdL15

L15 (60 mg, 0.00004377 mol) was dissolved in water (10 ml) to which Gd_2O_3 (7.9 mg, 0.00002188 mol) was added. The pH of the solution was raised from pH 5 to pH 8.5 by addition of LiOH (1M). The reaction was then heated to 80 ^{o}C for 24 hours. The cooled reaction was passed though a 0.5 μm filter and lyophilised, producing a white powder.

Yield 61.3 mg, 83.3%

CHN: Theoretical C 38.74, H 6.07, N 6.02. $C_{60}H_{92}N_8O_{24}Si_2Gd_2$

found C 38.78, H 6.04, N 5.94;

FTIR (KBr disk): 3427, 2957, 2849, 1592, 1418, 1248, 1102.

EuL15

L15 (60 mg, 0.00004377 mol) was dissolved in water (10 ml) to which Eu_2O_3 (7.7 mg, 0.00002188 mol) was added. The pH of the solution was raised from pH 5 to pH 8.5 by addition of LiOH (1M). The reaction was then heated to 80 °C for 24 hours. The cooled reaction was passed though a 0.5 µm filter and lyophilised, producing a white powder.

Yield 58.8 mg, 80.4%

FTIR (KBr disk): 3427, 2959, 2851, 1623, 1599, 1589, 1417, 1368, 1248, 1168, 1135.

CHN : Theoretical C 31.12, H 6.18, N = 4.83 . $C_{60}H_{92}N_8O_{24}Si_2Eu_2$

found C 31.06, H 5.51, N 4.78;

GdL16

L16 (60 mg, 0.0000297 mol) was dissolved in water (10 ml) to which Gd_2O_3 (5.4 mg, 0.0000148 mol) was added. The pH of the solution was raised from pH 5 to pH 8.5 by addition of LiOH (1M). The reaction was then heated to 80 °C for 24 hours. The cooled reaction was passed though a 0.5 µm filter and lyophilised, producing a white powder.

FTIR (KBr disk): 3399, 2360, 1596, 1419. 1315, 1249, 1085

Yield: 58.3 mg, 79.2%

CHN Theoretical C 28.84, H 5.81 N 4.41.

found C = 28.51, H = 5.21, N = 4.41;

EuL16

(60 mg, 0.0000297 mol) was dissolved in water (10 ml) to which Eu_2O_3 (5.2 mg, 0.0000148 mol) was added. The pH of the solution was raised from pH 5 to pH 8.5 by addition of LiOH (1M). The reaction was then heated to 80 °C for 24 hours. The cooled reaction was passed though a 0.5 µm filter and lyophilised, producing a white powder. Yield: 59.4 mg, , 81.2%

FTIR (KBr disk): 3414, 2964, 2927, 2857, 1589. 1414.30

CHN Theoretical C 40.60, H 5.75, N 6.52

found C 40.72, H 5.67, N 6.18;

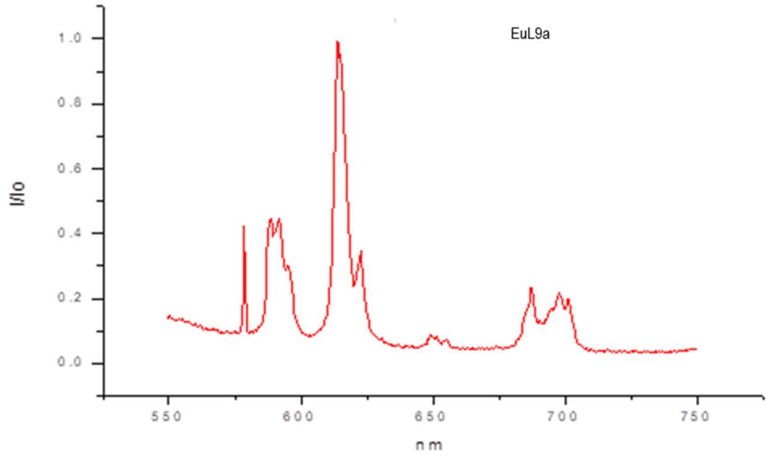
EuL9a
1.0
0.8
0.6
0.4
0.2
0.0
I/Io
550
600
650
700
750
nm

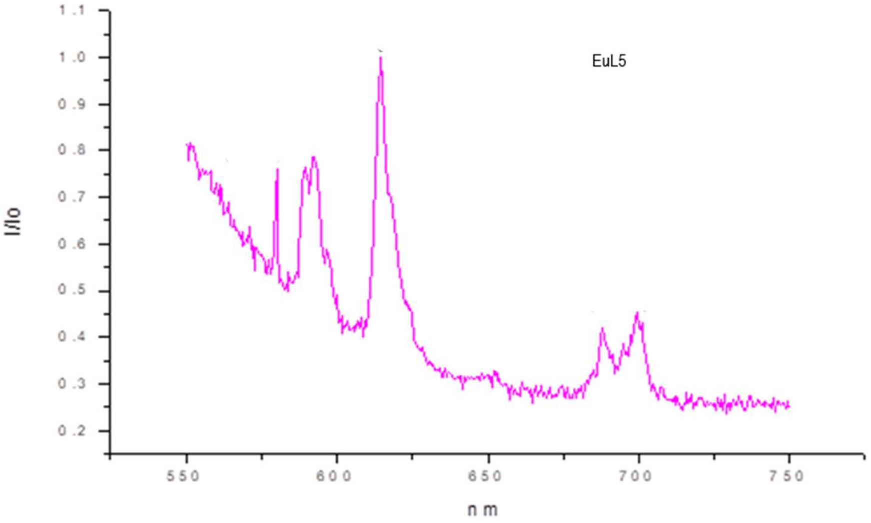
EuL5
1.1
1.0
0.9
0.8
0.7
0.6
0.5
0.4
0.3
0.2
I/Io
550
600
650
700
750
nm

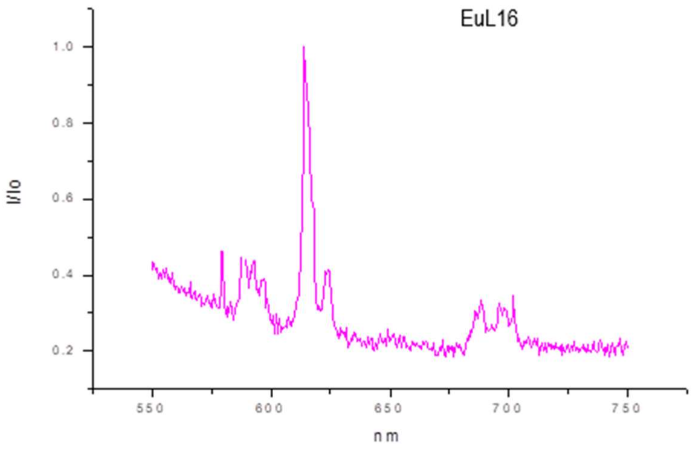

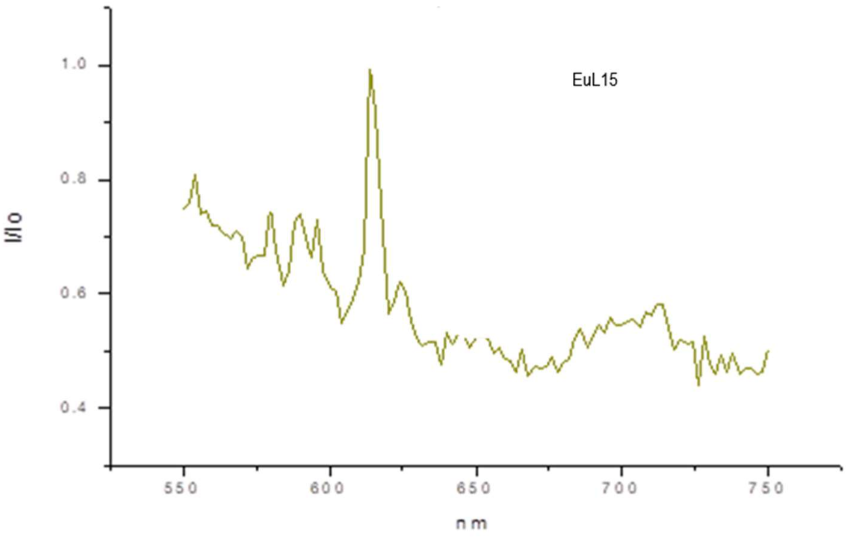

Relaxivity Measurements

Solutions of gadolinium complexes (0.95, 1.90, 2.85, 3.80, 4.75 mM) were prepared by dissolving the salt in ultra-pure water buffered to pH 7.5 using MOPS (10mM). For

accuracy, the solutions were made to volume in 5 ml volumetric flasks. A 5 mm NMR tube was then filled with solution and the tube placed in a Minispec mq60 pulsed time-domain analyser (Bruker, Massachusetts, USA). The temperature of the sample was maintained at 25 +/- 0.01 °C by an external circulator (Haake Phoenix II P1-C35P). Proton solvent longitudinal relaxation times (T_1) were measured for each sample at 60 MHz and 25 °C by means of the inversion-recovery technique. Three readings were taken for each sample and the average of the three readings represented the T_1 relaxation time of that sample. A plot of the inverse of the T_1 relaxation times versus the Gd^{3+} concentration gives the T_1-relaxivity (r_1) as the slope of the graph
